# What Ion Flow along Ion Channels Can Tell us about Their Functional Activity

**DOI:** 10.3390/membranes6040053

**Published:** 2016-12-13

**Authors:** Lucia Becucci, Rolando Guidelli

**Affiliations:** Department of Chemistry “Ugo Schiff”, University of Florence, Via della Lastruccia, 3, Sesto Fiorentino 50019, Italy; rolando.guidelli@libero.it

**Keywords:** bilayer lipid membrane, tethered bilayer lipid membrane, voltage-gated ion channel, ohmic ion channel, gramicidin, melittin, alamethicin

## Abstract

The functional activity of channel-forming peptides and proteins is most directly verified by monitoring the flow of physiologically relevant inorganic ions, such as Na^+^, K^+^ and Cl^−^, along the ion channels. Electrical current measurements across bilayer lipid membranes (BLMs) interposed between two aqueous solutions have been widely employed to this end and are still extensively used. However, a major drawback of BLMs is their fragility, high sensitivity toward vibrations and mechanical shocks, and low resistance to electric fields. To overcome this problem, metal-supported tethered BLMs (tBLMs) have been devised, where the BLM is anchored to the metal via a hydrophilic spacer that replaces and mimics the water phase on the metal side. However, only mercury-supported tBLMs can measure and regulate the flow of the above inorganic ions, thanks to mercury liquid state and high hydrogen overpotential. This review summarizes the main results achieved by BLMs incorporating voltage-gated channel-forming peptides, interpreting them on the basis of a kinetic mechanism of nucleation and growth. Hg-supported tBLMs are then described, and their potential for the investigation of voltage-gated and ohmic channels is illustrated by the use of different electrochemical techniques.

## 1. Introduction

The traditional and most widely employed procedure for investigating the functional activity of channel-forming peptides and proteins consists in using bilayer lipid membranes (BLMs) interposed between two aqueous solutions [1,2]. The functional activity is revealed by a flow of physiologically relevant inorganic ions, such as Na^+^, K^+^ and Cl^−^, across the BLM either from cyclic voltammetry (current-voltage curves in biophysical jargon) or chronoamperometry measurements (current-time curves) [3]. A major drawback of BLMs is their fragility, high sensitivity toward vibrations and mechanical shocks, and low resistance to electric fields; thus, they hardly last more than eight hours and collapse for potential differences between the solutions that bath the two sides of the BLM greater than 100–150 mV. To overcome this problem, metal-supported tethered BLMs (tBLMs) have been devised, where the BLM is anchored to the metal via a hydrophilic spacer that replaces and mimics the water phase on the metal side [3,4]. TBLMs are fabricated by tethering a “thiolipid” monolayer to the surface of a noble metal such as Au, Ag or Hg. Thiolipid molecules consist of a hydrophilic chain (the spacer) terminated at one end with a sulfhydryl or disulfide group for anchoring to the support and covalently linked at the other end to two alkyl chains simulating the hydrocarbon tails of a phospholipid. A tethered thiolipid monolayer exposes its hydrophobic lipid moiety on the opposite side with respect to the metal surface, thus providing one half of the lipid bilayer. The other half is formed by self-assembling a lipid monolayer on top of the thiolipid monolayer, exploiting the hydrophobic interactions between the alkyl chains of the thiolipid monolayer and those of the lipid monolayer.

Gold- and silver-supported tBLMs may provide useful structural information on the tBLM architecture and on how it is affected by protein incorporation, by the use of several surface sensitive techniques (e.g., polarization modulation infrared reflection-absorption spectroscopy, neutron reflectivity, atomic force microscopy, scanning tunneling microscopy, surface plasmon resonance spectroscopy) [5,6,7]. However, this structural information can seldom be directly related to the functional activity of the protein. Direct investigation of protein functional activity via inorganic ion flow along ion channels incorporated in Au- and Ag-supported BLMs is prevented by non-negligible water electroreduction at physiological pH and by a number of defects that provide a nonselective pathway to ion flow [8,9]. To the best of our knowledge, the only tBLMs allowing the functional activity of proteins to be directly monitored by measuring the protein-induced ion flow across the lipid bilayer are mercury-supported tBLMs [3,4,10]. Thanks to its liquid state, mercury provides the lipid bilayer with a perfectly smooth surface and a lateral mobility of the lipid molecules comparable with a BLM, healing surface defects; moreover, its high hydrogen overpotential eliminates water electroreduction at physiological pH. Mercury-supported tBLMs react to the presence of proteins, charges and physical forces in a dynamic and responsive manner, by reorganizing upon interaction with external perturbations and mimicking the functionality of living cell membranes. These biomimetic membranes can make space for relatively large membrane proteins, such as the HERG potassium channel [11], incorporating them from their detergent solutions; it is only required that at least one of the two extramembrane domains of the protein is small enough to be accommodated in the hydrophilic spacer. These membranes also determine the spontaneous separation of the components of a lipid mixture (“demixing”), giving rise to the formation of important lipid microdomains, called lipid rafts [12]. A drawback in the use of mercury-supported tBLMs is represented by the notable difficulty in using surface sensitive techniques for their structural characterization.

This review is organized as follows. The main results achieved by conventional BLMs incorporating voltage-gated channel-forming peptides are briefly summarized, and their interpretation on the basis of a kinetic mechanism of nucleation and growth is outlined. Subsequently, mercury-supported tBLMs are described, and their potential for the investigation of voltage-gated and ohmic channels is illustrated by the use of potential-step chronocoulometry, electrochemical impedance spectroscopy and cyclic voltammetry.

## 2. Bilayer Lipid Membranes (BLMs)

A BLM consists of a lipid bilayer that occludes a small hole, about 1 mm in diameter, in a Teflon septum that separates two aqueous solutions [1,2,3]. According to the so-called “painting method”, the bilayer is spontaneously formed by placing a drop of a lipid in a suitable solvent, e.g., decane, on the hole, using a brush. These BLMs provide a friendly environment to integral proteins, but incorporate an appreciable amount of molecules of the alkane used as solvent in the hydrocarbon tail region. A more sophisticated procedure for the preparation of solvent-free BLMs, devised by Montal and Mueller [1], allows the formation of solvent-free lipid bilayers that may also be composed of two different lipid monolayers. The differential capacitance of these solvent-free BLMs amounts to 0.8–0.9 μF·cm^−2^ and is higher than that, about 0.3–0.4 μF·cm^−2^, of BLMs obtained by the painting method. The presence of an organic solvent in the latter BLMs decreases their differential capacitance. BLMs of small surface area (1 × 10^−3^ cm^2^ or less) allow the recording of single channel currents under voltage-clamp conditions by means of a “patch-clamp” amplifier. BLMs have been extensively employed as matrixes for the incorporation of integral proteins, photoactive pigments and biomolecules involved in biophysical, biochemical and physiological studies. The electric potential difference between the two aqueous solutions that bath a BLM (i.e., the “transmembrane potential” *φ_m_*) is simply provided by the voltage applied between two identical reference electrodes placed in these two solutions.

### 2.1. Ohmic and Voltage-Gated Channels

Leaving aside ligand-gated ion channels, the other ion channels may be either ohmic or voltage-gated. Ohmic channels exhibit a plot of the average or single channel current against *φ_m_* that is linear across the zero transmembrane potential [13]. This linearity is maintained throughout the whole accessible *φ_m_* range if the two bathing solutions have identical composition. If the two solutions differ in their electrolyte concentration, progressive deviations from linear behavior are observed as we depart from the zero transmembrane potential, with the BLM opposing greater resistance to the flow of ions as *φ_m_* moves them from the lower to the higher concentration. Conversely, voltage-gated channels yield plots of the current against *φ_m_* that increases exponentially on one side of *φ_m_* = 0, while the current is vanishingly small on the other side [14,15]; in other words, they open or close in response to changes in the direction of the transmembrane potential. A typical example of ohmic channel is offered by gramicidin [13]. This unique channel-forming peptide has a helical structure that differs from the *α*-helix of common peptides and membrane proteins, in that its lumen is large enough to allow the passage of simple desolvated monovalent cations. Since its length is about one half that of a biomembrane, it spans the membrane by forming a dimeric channel, which is stabilized by the cation flow [16].

By far the majority of channel-forming peptides and proteins have an *α*-helical structure in a lipid environment and form ion channels by aggregation of monomeric units. One side of the *α*-helices is relatively hydrophilic by the presence of polar or charged residues and is turned toward the lumen of the aggregate, whereas the opposite side is hydrophobic and is turned toward the surrounding lipid molecules, where it interacts attractively with them. As a rule, the aggregation of *α*-helices with ion channel formation takes place by a kinetic mechanism of nucleation and growth. For these ion channels to exhibit an ohmic behavior, they must retain the same membrane-spanning structure in passing from one side of the zero transmembrane potential to the other. This situation is encountered only rarely. An example is provided by the lipodepsipeptide syringopeptin 25A (SP25A), whose single channel current vs. *φ_m_* plot is roughly linear over a narrow potential range straddling *φ_m_* = 0 [17]. However, this plot is obtained upon adding the peptide on one side of the BLM (the *cis* side), stepping *φ_m_* from positive values on the *trans* side (i.e., “trans-positive” values) to equal and opposite trans-negative values, and plotting the rate of change of the current with time as measured just before and after each voltage step against *φ_m_*. While the current at trans-positive potentials increases with time, that of opposite sign flowing as a consequence of the potential steps to the corresponding trans-negative values decays with time, becoming vanishingly small in about 10 s. This denotes an unstable state at trans-negative potentials, which determines a slow conformational change of the channel, accompanied by a movement of charge (the “gating charge”). Nonetheless, the cyclic voltammograms of SP25A at a Hg-supported tBLM show a perfectly ohmic behavior [18] (see Section 3.4). Particular pretreatments at non-physiological trans-negative potentials also induce an ohmic behavior lasting several hours in a typical voltage-gated channel such as melittin [19,20]. 

By far the majority of *α*-helical peptides yield voltage-gated ion channels, when incorporated into BLMs. Several molecular models for voltage-gated channels have been proposed, with particular emphasis on the alamethicin channel. The simplest model assumes that channel formation starts from a small aggregate of peptide monomers (the nucleus) that grows in diameter through the uptake of further monomers. The voltage dependent step is the reorientation of the monomers by the electric field from the membrane surface into its interior, where they span the whole hydrophobic region and aggregate with ion channel formation [21]. Several other more involved models for peptide aggregation with ion channel formation have been proposed (for a review, see [22]), but the majority of evidence seems to be in favor of the aforementioned model.

There are quite different views about the peptide orientation at zero transmembrane potential, based on different experimental data and supported by different calculations. An NMR investigation showed that alamethicin interacts primarily at the water/lipid interphase without significant insertion into the hydrocarbon tail region in the absence of voltage [23]. On the other hand, circular dichroism data in unilamellar vesicles [24] and infrared attenuated total reflection spectroscopy in multibilayer membranes [25] pointed to alamethicin incorporation into the lipid film. Site-directed spin-labeling studies showed that alamethicin is in a linear form and normal to the membrane plane, with its C-terminus in the aqueous region and the N-terminus in the membrane hydrocarbon tails, even in the absence of voltage [26]. The aforementioned uncertainty in alamethicin orientation at zero transmembrane potential is not clarified by computational approaches [27,28].

### 2.2. What Can Be Learnt from Current-Time Curves at BLMs

**Figure 1:**
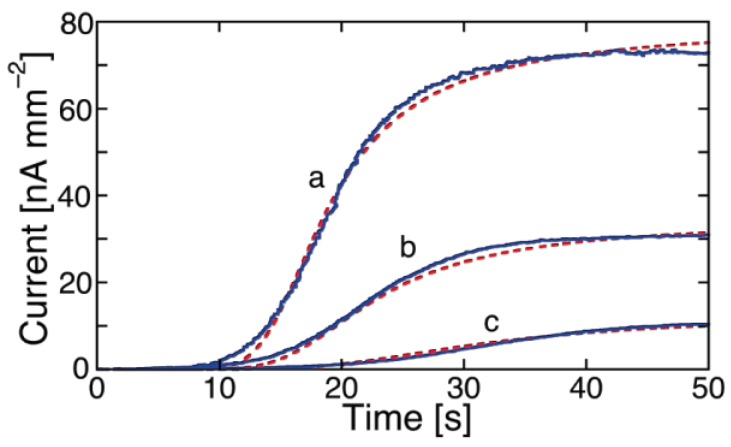
The solid curves are three successive current-time curves on the same bilayer lipid membranes (BLM) following transmembrane potential steps from 0 to −60 mV (a), −55 mV (b) and −50 mV (c), in aqueous 0.1 M KCl containing 0.625 μM monazomycin [29]. The corresponding dashed curves were calculated by the nucleation-and-growth model outlined in the text using the parameters *θ*_0_ = 0.1, *n* = 2, and *k_h_*_,*N*_*v_h_*_,*R*_^2^ = 0.016 s^−3^ for all three curves; *p* and *k_N_k_R_*^2^ were given the values: (a) 1 and 15 s^−3^; (b) 0.438 and 130 s^−3^; (c) 0.163 and 1250 s^−3^. The three calculated currents were matched to the experimental ones by multiplying them by the same factor 80. (Reprinted with permission from [30]. Copyright 2007, American Chemical Society).

The response of a conventional BLM incorporating a channel-forming peptide to a potential step is usually monitored by recording current-time (*I-t*) curves. This technique is referred to as *chronoamperometry* in electrochemical jargon. Peptides are often added only on one side of BLMs, referred to as the *cis* side. In what follows, the transmembrane potential will be defined as the electric potential on the *trans* side with respect to the *cis* side, taken conventionally equal to zero. Figure 1 shows *I-t* curves following transmembrane potential steps of increasing height at a BLM incorporating monazomycin [29]. The current induced by a voltage step rises in time approaching asymptotically a steady-state level, which increases with an increase in step height. This is due to an increase in the steady flow rate of permeant ions, elicited by the increase in the transmembrane potential.

Some peptides, such as alamethicin at ambient pressure [14,30], yield *I-t* curves that constantly maintain the concavity of the curve turned toward the time axis. Some others, such as monazomycin [29,31] and melittin [32], yield *I-t* curves that pass from being concave upward to concave downward, thus exhibiting a clear sigmoidal shape. Even alamethicin was reported to yield a sigmoidal shape under particular experimental conditions, such as an elevated pressure of 100 MPa [33] or during the recording of the first two or three *I-t* curves at a BLM freshly formed from a dioleoylphosphatidylcholine (DOPC) solution in decane [34]. Moreover, Mauro et al. [31], by carefully examining the *I-t* curve of alamethicin at ambient pressure in the range of a few tens of milliseconds from the start of the potential step, reported an initial S-shaped time course, followed by an asymptotic increase toward a steady-state value. It is, therefore, quite probable that the sigmoidal shape is a feature shared by all channel-forming *α*-helical peptides and ascribable to a common kinetic mechanism. If this kinetics is too fast to be resolved in the accessible time scale of potential-step experiments, then only *I-t* curves that are concave downward can be monitored, as in the case of the peptaibol alamethicin and of other Aib-containing peptides [35]. A three-state molecular model predicting a sigmoidal shape of current transients in response to potential steps was proposed by Bruner [36] by assuming that a peptide molecule moves, under the influence of an applied potential, first from a nonconducting surface state to a nonconducting precursor state and then to a conducting state. Current transients of sigmoidal shape for monazomycin [30] and potassium channels [37] were subsequently predicted by us on the basis a model accounting for monomer aggregation by a mechanism of nucleation and growth. Since this model also accounts for several properties of experimental current-voltage (*I-V*) curves of BLMs incorporating channel-forming peptides, we will now describe in some detail the main features of this modelistic approach [38]. 

Prior to a potential step inducing an *I-t* curve or a potential scan inducing an *I-V* curve, the monomers are regarded as located on the *cis* side of the membrane bathed by the solution where they were initially added. For the peptide dipolar molecule to span the hydrocarbon tail region of the BLM during a negative potential step or scan, the positive pole of the dipole will have to move across this region up to the attainment of the polar head region on the *trans* side, with the negative pole remaining on the *cis* side. The probability *p* of this charge movement depends critically upon the transmembrane potential.

Let us denote by *N_s_* and *N_t_* the number of peptide monomers located on the *cis* side of the membrane surface and with a transmembrane orientation, respectively. Under equilibrium conditions, their relative electrochemical potentials, ${\tilde{\mu }}_{s}$ and ${\tilde{\mu }}_{t}$, are equal for any value of the transmembrane potential *φ_m_*. These electrochemical potentials may differ not only by their electrical contribution, due to the different position of the positive pole within the membrane, but also by their chemical contribution *μ^θ^*, due to any conformational changes induced by such a charge movement. We can therefore write:

(1) μ˜s=μs0+kT ln Ns+qϕs=μ˜t=μt0+kT ln Nt+qϕt.

Here, *q* is the charge of the positive pole, which undergoes a shift from the *cis* to the *trans* side of the membrane, passing from a position where the transmembrane potential equals *φ_s_* to one where it equals *φ_t_*. Rearranging terms, we obtain:

(2) $$N_{t}/N_{s}=\text{exp}\;\left\{-\left[\Delta \mu^{\theta }+q\left(\phi_{t}-\phi_{s}\right)\right]/kT\right\}\;\text{with}:\;\Delta \mu^{\theta }\equiv \mu_{t}^{\theta }-\mu_{S}^{\theta }$$

Under the simplifying assumption that the peptide monomers are only allowed either to be located on the *cis* side of the membrane or to assume a transmembrane orientation, the probability *p* of their assuming the latter orientation is clearly given by *p* = *N_t_*/(*N_t_* + *N_s_*). Upon assuming for simplicity that the membrane spanned by the ion channel is homogeneous and that the electric field within it is constant, the electric potential varies linearly with distance. Denoting by *x_s_* and *x_t_* the distances of the charge *q* from the *cis* surface of the membrane in its initial *cis* and final *trans* locations, respectively, *φ*_s_ is given by *x_s_φ_m_*/*d*, and *φ_t_* by *x_t_φ_m_*/*d*, where *d* is the membrane thickness. Hence, *q*(*φ_t_* − *φ_s_*) equals *q*(*x_t_* − *x_s_*)*φ_m_*/*d* = ∆*mφ*_m_/*d*, where ∆*m* is the change in the dipole moment of the peptide as a consequence of its alignment along the direction of the electric field. Carrying out this substitution into Equation (2), the probability *p* is given by [3,38]:

(3) p=11+Ns/Nt=11+exp[Δmϕm/(dkT)]/a with: a≡exp(−Δμ0kT).

∆*μ^θ^* is the difference in Gibbs energy between the two conformational states of the peptide in the absence of the electric field (i.e., for *φ_m_* = 0). Since the parameter *a* is always much less that unity, it measures the probability *p* at zero transmembrane potential, in view of Equation (3).

As soon as a negative potential step or scan induces the peptide monomers to span the membrane with the *φ_m_*-dependent probability *p*, the resulting transmembrane monomers start nucleating, giving rise to channel-forming transmembrane clusters. Let *θ*_0_ denote the fraction of the whole BLM surface covered by the peptide molecules, irrespective of their orientation. Upon denoting by *S* the ratio of the area covered by transmembrane clusters to that covered by both transmembrane monomers and transmembrane clusters, the fraction of the whole BLM surface covered by the transmembrane monomers is given by *Θ* ≡ *θ*_0_*p*(1 − *S*), whereas *θ*_0_*pS* is clearly the fraction covered by the transmembrane clusters. Clusters of transmembrane monomers resulting from a series of consecutive collisions are characterized by a critical size (the “nucleus”), below which they have a higher tendency to shrink by releasing one unit than to grow by aggregation of a further unit, and above which they have a practically irreversible tendency to increase. The formation of this critical cluster size from embedded monomeric units is called “nucleation”, whereas the irreversible increase beyond the critical size is referred to as “growth”. If the nucleus is composed of a number *n* of transmembrane monomers, the elementary step yielding the nucleus consists of the incorporation of a monomer into a (*n* − 1)-meric “subcritical nucleus”. This results from (*n* − 1) elementary steps consisting in the incorporation of each monomeric unit into the immediately preceding subcritical nucleus, starting from an initial transmembrane monomer acting as a “nucleation center”. If all steps preceding the step yielding the nucleus are considered to be in quasi equilibrium, then the nucleation rate, *v_N_*, will be proportional to the *n*th power of the fractional surface coverage, *Θ* = *θ*_0_*p*(1 − *S*), by the transmembrane monomers randomly distributed in the lipid bilayer, according to a nucleation rate constant *k_N_*:

(4) d*N*/d*t* ≡ *v_N_* = *k_N_Θ^n^*

Here *N* is the number of nuclei per unit surface area. The irreversible aggregation of monomeric units to a nucleus, just after its formation, gives rise to a “supercritical nucleus”, whose continuous growth ultimately yields a channel-forming transmembrane cluster.

Upon assuming for simplicity that the cross-sectional area *A* of a growing supercritical nucleus is a circle of radius *R*, the rate of growth of *A* is given by the time derivative of *πR^2^*. This rate can be reasonably regarded as proportional to the frequency of the successful impacts of the transmembrane monomers, of surface coverage *Θ*, with the circumference *2πR* of the supercritical nucleus, according to a proportionality constant *k_R_*. Hence, we can write:

(5) d*A*/d*t* = d(*πR*^2^)/d*t* = 2*πR*d*R*/d*t* = *k_R_*2*πRΘ* → d*R*/d*t* ≡ v*_R_* = *k_R_Θ*

It follows that the rate *v_R_* of radial growth of a supercritical nucleus is proportional to *Θ* according to the rate constant *k_R_*. Successful impacts require a favorable mutual orientation between the supercritical nucleus and the aggregating transmembrane monomer, and are not necessarily controlled by the two-dimensional diffusion of the transmembrane monomers within the lipid bilayer.

Let us now consider a general approach to the kinetics of nucleation and growth that allows the quantity *S* to be calculated as a function of time. Upon setting equal to 0 the starting time of the nucleation and growth process, let us imagine observing it at a later time *t*. Let d*N* denote the infinitesimal number of nuclei that are forming in the infinitesimal time interval between *y* and *y* + d*y* before the observation time *t*. Having assumed that the resulting supercritical nuclei have a circular shape, the nuclei formed in this infinitesimal time interval make the following contribution to the area covered by the supercritical nuclei at time *t* [3,38]:

(6) $$\text{dN}\int_{0}^{A\left(t\right)}dA=\pi {\left(\int_{0}^{R}dR\right)}^{2}dN=\pi {\left[\int_{y}^{t}{\left(dR/dt\right)}_{z}dz\right]}^{2}dN$$

Here, *z* is an auxiliary variable that has the dimensions of time and varies between the time *y* at which the nuclei form and the time *t* at which the resulting supercritical nuclei are observed. If we now sum all the above infinitesimal contributions by integrating Equation (6) over time between the limits of integration *y* = 0 and *y* = *t*, we obtain the ratio of the area covered by the supercritical nuclei (ultimately yielding the channel-forming clusters) to that originally covered by all monomers, irrespective of their orientation [39]:

(7) $$S_{x}=\pi \int_{0}^{t}dy{\left[\int_{y}^{t}{\left(dR/dt\right)}_{z}dz\right]}^{2}{\left(dN/dt\right)}_{y}=\pi \int_{0}^{t}dy{\left[\int_{y}^{t}v_{R}\left(z\right)dz\right]}^{2}v_{N}\left(y\right)$$

This ratio is denoted by *S_x_*, rather than *S*, to emphasize that it ignores the possible overlapping of the progressively growing supercritical nuclei; it is commonly referred to as the “extended area”. However, strictly speaking, *S_x_* is not the” extended equivalent” of *S*. In fact, *S* is the ratio of the actual area covered by the supercritical clusters to that covered by both supercritical clusters and transmembrane monomers, thus ignoring the monomers located on the *cis* side of the membrane. In this respect, *S_x_* corresponds to the “extended equivalent” of the product *pS*, and not of *S*. The expression in Equation (7) includes both the rate of nucleation, (d*N*/d*t*) = *v_N_*, and the rate of radial growth, (d*R*/d*t*) = *v_R_*. Being entirely general, it can be applied to the kinetics of any nucleation-and-growth process.

The unrealistic overlapping of the progressively growing supercritical nuclei can be avoided by making use of Avrami’s formalism [40]. Roughly speaking, Avrami’s approach relies on the consideration that the area covered by the growing supercritical nuclei is an extensive property of the system and, as such, is directly proportional to the “available area”. If, *ab absurdo*, this concept is applied to the case in which the single growing supercritical nuclei are allowed to grow without being limited by the neighboring supercritical nuclei, then the available area is the total surface area, *S*_T_, where the nucleation-and-growth process occurs, and we will write:

(8) d*S_x_*/d*t* = const. × *S_T_*

*S_x_* is just the aforementioned hypothetical extended area, namely the area that would be covered by all the growing supercritical nuclei if they were free to grow without limits. This is not possible in reality, and the actual surface area available to the growing supercritical nuclei is that still uncovered, which is given by the difference, *S_T_* − *S*, between the total area, *S_T_*, and that, *S*, already covered. In this case, Equation (8) will become:

(9) d*S*/d*t* = const. × (*S_T_* – *S*)

Upon eliminating the common proportionality constant between Equations (8) and (9), we obtain [2]:

(10) $$dS/dt=\left(1-S/S_{T}\right)dS_{x}/dt\;or,\;for\;S_{T}=1:\;dS/dt=\left(1-S\right)dS_{x}/dt$$

The present model of nucleation and growth is entirely general. Thus, it only assumes that the elementary steps preceding the step yielding the nucleus are in quasi equilibrium, and that the growth of the supercritical nuclei proceeds irreversibly by activated aggregation of transmembrane monomers. The expressions of Equations (4) and (5) for the rates, *v_N_* and *v_R_*, of nucleation and radial growth must be substituted into the expression of Equation (7) for the extended area *S_x_*. In doing so, we must consider that *S_x_* is the extended equivalent of *pS*. Hence, in carrying out these substitutions, the complement of *S* to unity, (1 − *S*), must be multiplied by *p* in the quantity *Θ* = *θ*_0_*p*(1 − *S*), for consistency [41], yielding:

(11) $$S_{x}=\pi \int_{0}^{t}dy{\left[\int_{y}^{t}k_{R}p\Theta \left(z\right)dz\right]}^{2}k_{N}{\left(p\Theta \left(y\right)\right)}^{n}=\pi k_{R}^{2}k_{N}\theta_{0}^{2+n}p^{4+2n}\int_{0}^{t}dy{\left[\int_{y}^{t}\left(1-S\left(z\right)\right)dz\right]}^{2}{\left(1-S\left(y\right)\right)}^{n}$$

By repeatedly differentiating Equation (11) with respect to time via the generalized Leibnitz formula, three differential equations are obtained. Combining these equations with the differential Equation (10) relating the extended area *S_x_* to the corresponding actual area *S*, a set of four differential equations is obtained, which can be readily solved numerically by the fourth-order Runge-Kutta method, yielding *S_x_* and *S* as a function of time. It is worth noting that the kinetics of nucleation and growth depends exclusively on the product, *k_N_k_R_*^2^, of the rate constant of nucleation by the square of the corresponding rate constant of radial growth, thus reducing the number of adjustable parameters to only four, i.e., *p*, *θ*_0_, *n*, and *k_N_k_R_*^2^.

Finally, the current density *j* across the BLM induced by the transmembrane-potential jump is obtained by setting it proportional to the fractional surface coverage by the channel-forming transmembrane clusters, *θ*_0_*pS*, since each newly formed channel makes a contribution to this current:

(12) $$j\;\propto \;\theta_{0}pS$$

The limiting value attained by *j* is clearly given by *θ*_0_*p*, since *S* will ultimately tend to unity.

The polyene-like antibiotic monazomycin incorporated in a BLM yields sigmoidal *I-t* curves [29], as shown in Figure 1. However, the typical sigmoidal current rise is preceded by a relatively long period of time during which no ion flow across the BLM occurs. The resulting long “foot” of the current transients is to be ascribed to the presence of peptide aggregates flatly adsorbed on the BLM surface at the initial potential. These flat clusters have the polar/charged side chains turned toward the aqueous phase and the hydrophobic side chains turned toward the interior of the clusters, with an arrangement opposite to that in the ion channels. Hence, their incorporation into the BLM following the potential step requires their previous disruption into flat monomers, to be converted into transmembrane monomers. The kinetic process of disruption of flat clusters can be formally treated as a nucleation of holes and growth of hole aggregates within the clusters. By nucleation of holes, we mean the quasi-reversible detachment of an initial number of flat monomers from a flat cluster and their random intercalation with the water molecules on top of the BLM. In other words, a hole is just a flat monomer that, upon detachment from a flat cluster, leaves behind a hole in the cluster. These flat monomers are considered to detach from a flat cluster and to re-aggregate into it in a quasi-reversible manner, until the number of nearest-neighboring holes in the cluster attains a critical value (*n*) beyond which this number increases irreversibly up to complete disruption of the flat cluster. This critical number of nearest-neighboring holes can again be loosely regarded as a nucleus. Nucleation is followed by the irreversible disruption of the flat clusters, which can be viewed as an irreversible “growth of hole aggregates”, according to the nucleation-and-growth terminology. This disruption is triggered by the potential step, which starts stripping the flat monomers (the holes) from the flat clusters by dragging them into the BLM as transmembrane monomers. The rate constant of nucleation of holes, *k_h_*_,*N*_, and the rate of radial growth of hole aggregates, *v_h_*_,*R*_, are not directly affected by the electric field. However, they depend indirectly upon the electric potential via the penetration probability *p* of the flat monomers. In Figure 1, the experimental *I-t* curves of monazomycin are fitted by a model that combines the nucleation of holes and growth of hole aggregates with the nucleation of transmembrane monomers and growth of transmembrane supercritical nuclei [30].

### 2.3. What Can Be Learnt from Current-Voltage (I-V) Curves at BLMs

*I-V* curves at BLMs are obtained by scanning the applied electric potential between an initial and a final value at a constant scan rate *v*. The electric potential is usually applied between two identical reference electrodes immersed in the two aqueous solutions that bath the two sides of the BLM. This potential, which is just the transmembrane potential *φ_m_*, is a thermodynamically significant quantity and is referred to as “voltage” (*V*) in biophysical jargon. The voltage may also be scanned back and the resulting voltage cycle may be repeated several times, a procedure referred to as “cyclic voltammetry” in electrochemical jargon; in this case the current is plotted versus the voltage back and forth to yield the cyclic voltammetry trace. When the peptide is added on only one side of the BLM, identified with the *cis* side, the voltage *V* will be referred to the *trans* side with respect to the *cis* side, taken as zero. This definition, which is the opposite of that adopted in the biophysical literature, complies with that adopted at metal-supported tBLMs, where the applied potential *E* is always referred to the metal with respect to the solution. Accordingly, the current will be taken as negative when cations move from the *cis* to the *trans* side, at variance with the biophysical usage.

**Figure 2:**
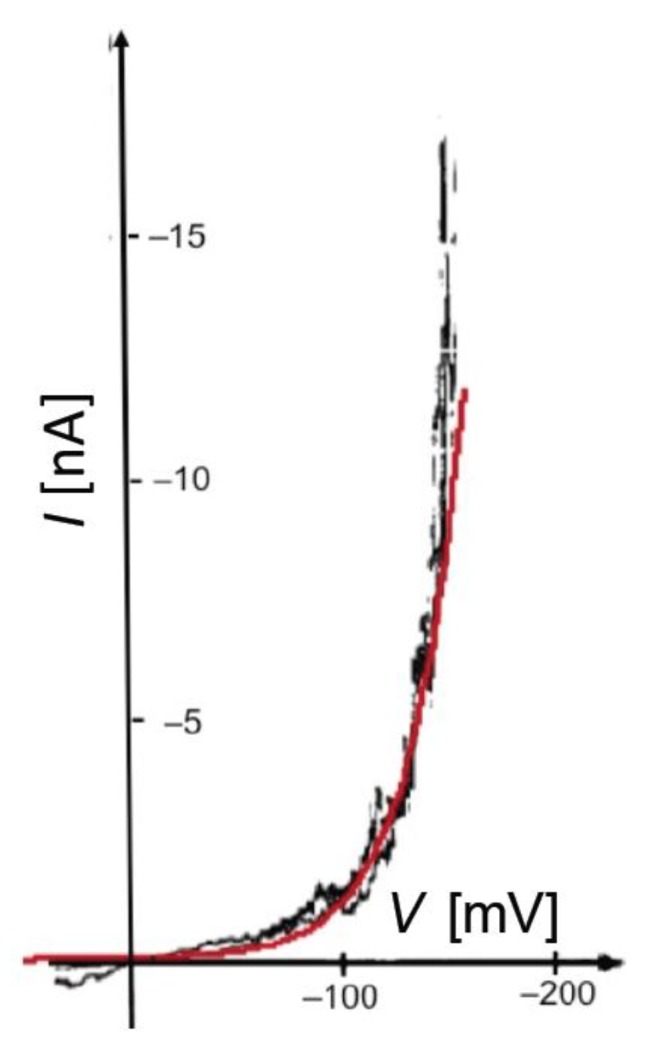
Experimental *I-V* curve for 0.4 μg/mL melittin in dioleoylphosphatidylcholine (DOPC), taken directly from Figure 7 of Pawlak et al. [20] (black curve), and curve calculated for *a* = 1 × 10^−4^, ∆*m* = 70 D, *θ*_0_ = 0.1, *k_N_k_R_*^2^ = 1 × 10^6^ s^−3^ and *n* = 1 (red curve). The height of the calculated curve was normalized to that of the experimental one. (Reprinted with permission from [38]. Copyright 2015, Elsevier).

In BLMs incorporating channel-forming peptides from only the *cis* side, a negative voltage scan induces an exponential increase in the negative current, as shown in Figure 2 for a BLM incorporating melittin. Thus, the slope of the resulting *I-V* curve, which measures the conductance *G*, is such that its logarithm increases linearly with an increase in the absolute value, |*V*|, of the voltage. An increase in the concentration of a channel-forming peptide in the bathing solution shifts the *I-V* curve toward less negative *V* values. At constant conductance, |*V*| decreases linearly with the logarithm of the peptide concentration.

Alamethicin and melittin are the two most thoroughly investigated voltage-gated channel-forming peptides in BLMs. In spite of their structural differences, they have several functional features in common. Thus, under suitable experimental conditions, they both exhibit two distinct relaxation processes, a fast relaxation process with weakly voltage-dependent conductance, and a slow relaxation one with strongly voltage-dependent conductance. In particular, this situation is encountered when the peptide is added to only the *cis* side of the membrane, and the current flows only in the direction from the *cis* to the *trans* side, provided the voltage is sufficiently negative. The conductance *G* associated with any of the two different relaxation processes depends upon *V* and the peptide concentration, *c_pep_*, according to the equation [22,42]:

(13) $$G\;\propto \;c_{pep}^{\delta }\text{exp}\left(-\gamma FV/RT\right)$$

The fast relaxation process has a time constant two orders of magnitude smaller than that of the slow relaxation one and occurs in the millisecond range, which is of the same order of magnitude as the mean lifetime of single pore states [34]; it appears to arise from a shift in the probability distribution of the different conductance levels of a pore as the voltage is changed. Conversely, the slow relaxation process is attributed to the fluctuation in the number of channels present in the membrane [34,43] and correlates well with the lifetime of single current bursts. The conductance of the fast relaxation process is from one to two orders of magnitude smaller than that of the slow relaxation process. The passage from a weakly to a strongly voltage-dependent conductance with a progressive negative shift in voltage is shown by a number of synthetic Aib-based polypeptides of different length incorporated into palmitoyloleoylphosphatidylcholine (POPC) BLMs [35]. The critical voltage marking the passage from a weakly to a strongly voltage-dependent conductance regime is accompanied by a dramatic rise of current and shifts toward more negative values with a decrease in the polypeptide chain length and in its concentration in the bathing solution.

During the slow relaxation process, alamethicin yields *γ* values ranging from 4.4 to 6 and *δ* values ranging from 6 to 11, depending on the lipid composition of the BLM [34,42], whereas melittin yields *γ* = 4.3 and *δ* ≈ 4 [32] and synthetic Aib-based peptides yield *γ* = 4.7 and *δ* = 8.3 [35]. Conversely, during the fast relaxation process, alamethicin yields *γ* = 0.96 and *δ* ≈ 2 [34], synthetic Aib-based peptides yield *γ* values ranging from 2.8 to 3.3 and *δ* ≈ 2 [35], whereas melittin yields *γ* values ranging from 1.3 to 1.6 and *δ* ≈ 4 [32], although a *δ* value of 2.5 at zero voltage was also reported [44]. The *δ* parameter for melittin was found to decrease from ~2 to ~0.5 with a decrease in the chain length of the lipid molecules forming monoglyceride/squalene BLMs [45]. Summarizing, the dependence of the current upon the peptide concentration increases by several orders of magnitude in passing from the fast to the slow relaxation process.

When melittin is added on the *cis* side of the BLM, the steady-state *I-V* curve shows both a positive and a negative branch, albeit not perfectly symmetrical with respect to *V* = 0 [20]. Adding alamethicin on the *cis* side yields a single negative branch of the *I-V* curve if the BLM is formed with a saturated lipid [14,46]. On the other hand, with unsaturated or halogenated membrane lipids, alamethicin yields both branches, with the positive branch more shifted with respect to zero voltage than the negative one [46]. This suggests that the presence of saturated lipids in the BLM hinders the diffusion of alamethicin across the membrane.

The salient features of the experimental behavior of voltage gated channels at BLMs can be accounted for by the same general approach outlined in Section 2.2, which treats the time-dependent formation of ion channels as a nucleation of transmembrane peptide monomers and growth of the resulting supercritical nuclei. The probability *p* for the passage from a flat to a transmembrane orientation is again expressed by Equation (3). Being a function of the transmembrane potential *φ_m_* = *V*, *p* is time independent after the instantaneous voltage step in *I-t* measurements, whereas it varies with time in *I-V* measurements. Nonetheless, even in the latter case, the probability *p* must be removed from under the integral signs in the expression of *S_x_*, as done in Equation (11). This removal is necessary, since *p* is an implicit function of time *t* and is operative from the starting time set equal to 0, thanks to the constant voltage scan rate *v* ≡ d*V*/d*t*. Conversely, the quantity *S_x_* is a function of the integration variables *z* and *y* and refers to the events occurring throughout the various nucleation processes starting at different times *y* and followed by the growth of the resulting supercritical nuclei, up to the final observation time *t*.

The last step consists in calculating the current density *j* from the absolute rate theory of ion transport across membranes [47], as applied to ion translocation across the potential energy barrier located in the hydrocarbon tail region of the BLM. In applying this equation, the potential energy barrier will be regarded as symmetrical and nonselective toward ion flow, for simplicity, by ascribing a common value, *k_t_*, to the forward and backward rate constants at zero voltage. Finally, the ionic current density *j* across the membrane, which would be equal to zero in the absence of ion channels, is set proportional to the fractional surface coverage by the embedded channel-forming clusters, *θ*_0_*pS*, since each newly formed channel makes a contribution to this current. With the above assumptions, the current density *j* expressed by the absolute rate theory becomes:

(14) $$j=\left(\theta_{0}pS\right)Fk_{t}\left[\left(-c_{cis}^{+}e^{-\frac{\alpha FV}{RT}}+c_{trans}^{+}e^{\frac{\left(1-\alpha \right)FV}{RT}}\right)+\left(c_{cis}^{-}e^{\frac{\alpha FV}{RT}}-c_{trans}^{-}e^{-\frac{\left(1-\alpha \right)FV}{RT}}\right)\right]$$

Here, *α* is the transfer coefficient, *c^+^_cis_*, *c*^+^*_trans_* are the concentrations of a monovalent cation on the *cis* and *trans* sides of the membrane, and *c*^−^*_cis_*, *c*^−^*_trans_* are those of a monovalent anion. The first and second expressions between parentheses in Equation (14) measure the cation and anion currents, respectively.

Upon setting for simplicity *c^+^_cis_* = *c^+^_trans_* = *c^−^_cis_* = *c^−^_trans_* ≡ *c*, *j* turns out to be proportional to *c*, besides being proportional to *k_t_*. The behavior of the dimensionless quantity *j*/(*Fk_t_c*) will serve to show the way in which the quantities ∆*m*, *θ*_0_, *a* and the kinetic parameters of nucleation and growth affect the shape of *I-V* curves and the dependence of ln *G* upon *V* and the concentration, *c_pep_*, of the peptide in the bathing solution. Strictly speaking, setting the current density proportional to the number density of the embedded clusters that have undergone a kinetic process of nucleation and growth with ion channel formation amounts to assuming that only a single type of aggregate develops during the whole voltage scan. However, this assumption is also valid if the single-channel state distributions are independent of voltage and the macroscopic conductance induced by the peptide exclusively arises from the increase in the number of channels as |*V*| increases. These requirements are approximately satisfied by the alamethicin channel in a phosphatidylethanolamine BLM [14] and by melittin in a DOPC BLM [20].

**Figure 3:**
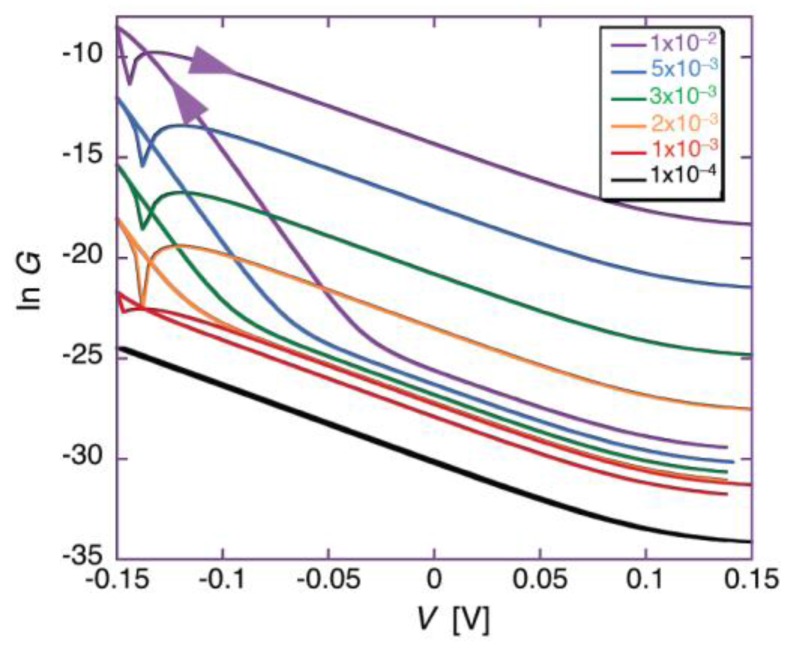
Plots of ln *G* vs. *V* for different values of *a* and for ∆*m* = 70 D, *θ*_0_ = 0.1, *k_N_k_R_*^2^ = 1 × 10^6^ s^−3^ and *n* = 1. The curves shift toward progressively lower ln *G* values with decreasing *a*. (Reprinted with permission from [38]. Copyright 2015, Elsevier).

Figure 3 shows plots of ln *G* against *V* for different values of the probability, *a*, of the peptide dipoles being in the transmembrane orientation at zero voltage, and for ∆*m* = 70 D, *θ*_0_ = 0.1, *k_N_k_R_*^2^ = 1 × 10^6^ s^−3^ and *n* = 1. All plots refer to a temperature of 298 K, a voltage scan rate of 10 mV/s and a membrane thickness *d* = 30 Å. The plots show ln *G* both along the negative-going voltage scan and along the subsequent positive-going one. The sharp dip marks the maximum negative current attained at the beginning of the reverse voltage scan.

**Figure 4:**
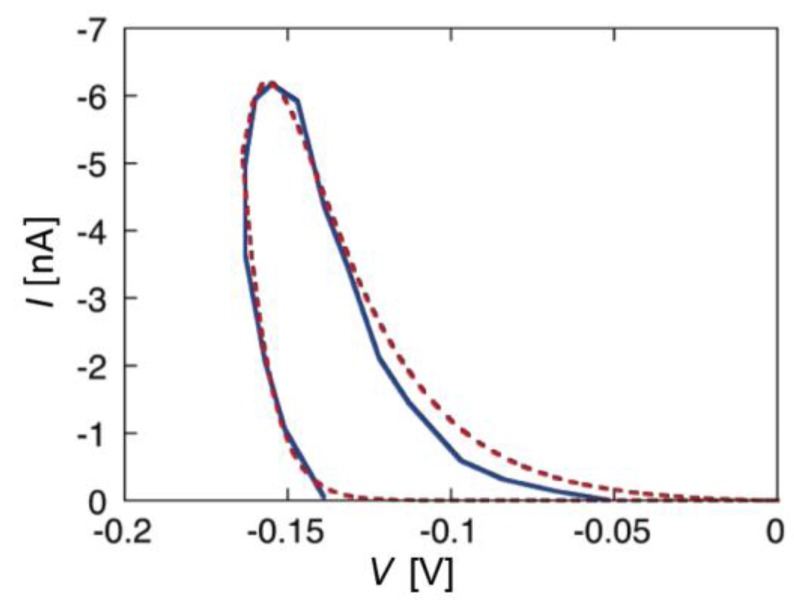
Experimental *I-V* curve for 0.2 μg/mL alamethicin in bacterial phosphatidylethanolamine, taken from curve 2 in Figure 5 of Vodyanoy et al. [46] (blue curve) and curve calculated for *a* = 1 × 10^−2^, ∆*m* = 70 D, *θ*_0_ = 0.1, *k_N_k_R_*^2^ = 1 × 10^5^ s^−3^ and *n* = 1 (red curve). The height of the calculated curve was normalized to that of the experimental one. (Reprinted with permission from [38]. Copyright 2015, Elsevier).

During the negative-going voltage scan, the plots exhibit a region of weakly voltage-dependent conductance, with *γ* = 0.95, at lower negative voltages, as well as a region of strongly voltage-dependent conductance, with *γ* = 3.85, at higher negative voltages. At extreme positive voltages, *G* tends to attain a voltage-independent minimum value. During the positive-going voltage scan, the voltage dependence of the conductance is characterized by a *γ* value of 0.95, other than in the proximity of the voltage reversal. A progressive decrease of *a* lowers the ln G vs. *V* curve and gradually shifts its “elbow” toward more negative voltages. This causes the region of weakly voltage-dependent conductance to cover the whole negative voltage range usually spanned by experimental *I-V* curves. As a result of this trend, the *I-V* curves falling over this voltage range and calculated for *a* values varying from 1 × 10^−2^ to 5 × 10^−3^ show a strongly voltage-dependent conductance and an appreciable hysteresis, whereas those with *a* < 2 × 10^−3^ show a weakly voltage-dependent conductance and a small or negligible hysteresis. This behavior is exemplified by the calculated *I-V* curves in Figure 2 and Figure 4, which refer to the two different situations. Figure 4 shows an experimental *I-V* curve for 0.2 μg/mL alamethicin in a phosphatidylethanolamine BLM [46] and a corresponding curve calculated for *a* = 1 × 10^−2^, ∆*m* = 70 D, *θ*_0_ = 0.1, *k_N_k_R_*^2^ = 1 × 10^5^ s^−3^ and *n* = 1. The shape of the calculated curve closely simulates those reported for alamethicin [45,46,48] and for synthetic Aib-based polypeptides [35]; the *γ* value of 3.85 is also close to those for the synthetic polypeptides, although that for alamethicin is somewhat higher [34,35,42]. Conversely, Figure 2 shows an experimental *I-V* curve for 0.4 μg/mL melittin in a DOPC BLM [20] and a corresponding curve calculated for *a* = 1 × 10^−4^, ∆*m* = 70 D, *θ*_0_ = 0.1, *k_N_k_R_*^2^ = 1 × 10^6^ s^−3^ and *n* = 1, in which the forward and backward voltage scans practically coincide.

The experimental *I-V* curves for alamethicin and melittin in Figure 2 and Figure 4 being simulated with *a* = 1 × 10^−2^ and 1 × 10^−4^, respectively, is consistent with their different structures. Thus, if both peptides are incorporated in the *cis* polar head region of the BLM at positive voltages, the Gibbs energy required for a negative-going voltage scan to reorient the peptide dipoles from a flat to a transmembrane stand is expected to be higher for melittin than for alamethicin. In fact, the Gibbs energy required to push the positively charged N-terminal amino group and the Lys-7 residue of the N-terminal sequence of melittin into the hydrocarbon tail region is much greater than that required to push the neutral N-terminal of the alamethicin molecule. The regions of weakly and strongly voltage-dependent conductance in the ln *G* vs. *V* curves of Figure 3 are also in semiquantitative agreement with the corresponding fast and slow relaxation processes exhibited by the current-time curves in voltage step experiments, characterized by *γ* ≈ 0.96 and 6.4 for alamethicin [34], and by *γ* ≈ 1.3 and 4.3 for melittin [32].

The *γ* values for the regions of weakly and strongly voltage-dependent conductance are only slightly affected by a change in the combined rate, *k_N_k_R_*^2^, of nucleation and growth. The main effect of an increase in *k_N_k_R_*^2^ from 10 to 1 × 10^7^ s^−3^, for *a* = 10^−2^ and the other parameters as in Figure 3, consists in a shift of the elbow of the bent ln *G* vs. *V* curves toward less negative voltages [38]. The effect of a decrease in the number of peptide monomers composing the critical nucleus from *n* = 4 to *n* = 1, with the other parameters as in Figure 3, is qualitatively similar to that produced by an increase in *k_N_k_R_*^2^. Thus, apart for the obvious changes in the absolute value of ln *G* with varying the adjustable parameters of the model, the slope of ln *G* against *V* yields *γ* values in fairly good agreement with the experimental ones over both regions of strongly and weakly voltage-dependent conductance, irrespective of the values ascribed to the adjustable parameters.

In the regime of strongly voltage-dependent conductance, the *γ* value predicted by the model increases linearly with the magnitude of the dipole moment and is expressed by the equation: *γ* = 0.66 + 0.044 Δ*m* [*D*], for *θ*_0_ = 0.1, *a* = 1 × 10^−2^, *k_N_k_R_*^2^ = 1 × 10^7^ s^−3^ and *n* = 1 [38]. Hence, the model predicts a *γ* value of 4, close to that reported for several channel-forming *α*-helical peptides, when ∆*m* is close to 70 D, which is the value estimated for an *α*-helical peptide spanning a hydrocarbon tail region 30 Å thick [22]. This strongly suggests that the change, ∆*m*, in the dipole moment normal component undergone by these peptides during a negative-going voltage scan is determined by the movement of their *α*-helix from the *cis* polar head region, with an orientation parallel to the membrane plane, to one spanning the hydrocarbon tail region (about 30 Å in thickness for a typical BLM). The above connection between the effective dipole moment of the peptide and the *γ* value has contributed to regarding *γ* as an “apparent gating charge”.

Along the region of weakly voltage-dependent conductance, the model predicts a *γ* value about equal to unity. This is due to the fact that over this region the ratio, *S*, of the number of peptide molecules aggregated into ion channels to the total number of molecules spanning the lipid bilayer is very low and almost voltage independent. Under these conditions, the voltage dependence of the current density *j* is only expressed by the product of *p* by the term between square brackets in Equation (14), and leads to a ln *G* vs. *V* plot of slope very close to unity. Since the kinetics of nucleation and growth exclusively affects the parameter *S*, and this is very small and roughly constant, the current is clearly insensitive to the *k_N_k_R_*^2^ and *n* values. Moreover, for *S* practically constant, the current turns out to be proportional to the fraction, *θ*_0_, of the membrane unit area covered by the peptide. Within the limits in which *θ*_0_ can be regarded as proportional to the peptide concentration, *c_pep_*, in the aqueous solution, the current along the low conductance region is proportional to *c_pep_*, and the parameter *δ* in Equation (13) equals unity. This prediction is consistent with the experimental observation that the low *γ* values characterizing the regime of weakly voltage-dependent conductance are often associated with *δ* values ranging from 1 to 2.5 [34,35,44], with the exception of melittin in asolectin BLMs [32,49]. It must be pointed out that the prediction of a *γ* value of about 0.95 along the region of weakly voltage-dependent conductance results from a passage of the peptide dipoles from a parallel to a vertical orientation with respect to the membrane plane as voltage becomes progressively more negative, similarly to what happens along the region of strongly voltage-dependent conductance. However, in this case no aggregation of transmembrane monomers occurs. For a typical dipole moment of 70 D, the mere dipole reorientation without aggregation yields a *γ* value of about 0.95. It is the aggregation of transmembrane dipoles with nucleation-and-growth kinetics that causes a significant *γ* increase from about 1 to about 4, when passing from the regime of weakly to that of strongly voltage-dependent conductance. This suggests that, in the regime of weakly voltage-dependent conductance, the ion movement elicited by the electric field occurs within a bunch of transmembrane monomers, whose side chains are randomly distributed with respect to each other (i.e., without aggregation into a proper ion channel).

The dependence of the conductance upon the peptide concentration predicted by the model in the regime of strongly voltage-dependent conductance is higher than that in the regime of weakly voltage-dependent conductance, as expressed by *δ* = 1. Thus, the plot of ln *G* against ln *θ*_0_ obtained from *I-V* curves calculated for ∆*m* = 70 D, *a* = 1 × 10^−2^, *k*_N_*k*_R_^2^ = 1 × 10^6^ s^−3^ and *n* = 1, i.e., under conditions of strongly voltage-dependent conductance, is linear and exhibits a slope of 3.85 over the whole *θ*_0_ range from 0.1 to 1 [38]. This value can be identified with *δ*, if we can reasonably assume that the peptide is incorporated from the bathing solution into the *cis* polar head region of the membrane according to Henry’s adsorption isotherm. This *δ* value is close to those reported in the regime of strongly voltage-dependent conductance for certain Aib-based polypeptides [35] and for melittin [32], whereas alamethicin exhibits appreciably higher values ranging from 6 to 11 [42]. The fact that the sole introduction of a mechanism of nucleation and growth for monomer aggregation into ion channels yields a *δ* value close to 4 as a natural consequence, demonstrates the inconsistency of the frequent assumption [20,50] that such a value is indicative of the formation of a tetrameric ion channel. The *δ* value for alamethicin being appreciably higher than predicted by the model, especially in certain BLMs, may possibly be ascribed to the failure of the assumption of voltage independence of the single-channel state distributions, on which Equation (11) relies. A higher *δ* value is expected if an increase in negative voltage tends to favor higher aggregates at the expense of lower ones.

## 3. Mercury-Supported Tethered Bilayer Lipid Membranes (tBLMs)

**Figure 5:**
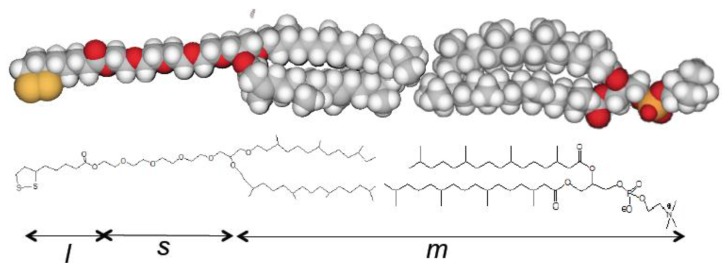
Space filling model and structure of the DPTL thiolipid lipid and of DPhPC, in a tail-to-tail configuration; *l*, *s*, and *m* denote the lipoic acid residue, the tetraethyleneoxy spacer and the hydrocarbon tail region, forming the monomeric unit of a DPTL/DPhPC tethered bilayer lipid membrane. Carbon atoms are in gray, hydrogen atoms in white, oxygen atoms in red, phosphorus atoms in orange and sulfur atoms in yellow.

Among the thiolipids tested in our laboratory, the one providing by far the most satisfactory results consists of a tetraethyleneoxy (TEO) hydrophilic chain, terminated at one end with a lipoic acid residue for anchoring to the metal surface, and covalently linked at the other end to two phytanyl chains mimicking the hydrocarbon tails of a lipid [51,52]. This thiolipid, 2,3,di-*O*-phytanyl-*sn*-glycerol-1-tetraethylene-glycol-d,l-*α* lipoic acid ester, is usually denoted by the acronym DPTL (2,3,di-*O*-phytanyl-*sn*-glycerol-1-tetraethylene-glycol-d,l-*α* lipoic acid ester lipid), where DP stands for diphytanoyl, T for tetraethyleneoxy and L for lipoic acid residue. Figure 5 shows the schematic picture of a DPTL molecule, with a diphytanoylphosphatidylcholine (DPhPC) monolayer on top of it.

Mercury-supported DPTL/phospholipid tBLMs are readily prepared by immersing a hanging mercury drop electrode (HMDE) into an ethanol solution of DPTL for about 20 min to anchor a thiolipid monolayer on the mercury surface. Incidentally, the time required to attain the same result on a gold surface amounts to 12–20 h. A phospholipid monolayer is then self-assembled on top of the thiolipid monolayer by simply immersing the thiolipid-coated mercury drop in an aqueous electrolyte on whose surface a lipid film has been previously spread [10,16]. Thanks to the hydrophobic interactions between the alkyl chains of the thiolipid and those of the lipid, this simple procedure gives rise to a lipid bilayer anchored to the mercury surface via the hydrophilic spacer moiety of the thiolipid. By avoiding the use of vesicles, this procedure excludes any artifacts due to partially fused vesicles. The hydrophilic spacer moiety of the thiolipid may accommodate ions capable of translocating across the lipid bilayer with the aid of an ion channel or carrier, thus acting as an “ionic reservoir”. These advantageous features make the incorporation of membrane proteins in mercury-supported thiolipid-based tBLMs easier and safer than in solid-supported tBLMs.

### 3.1. What Can Be Learnt from Potential-Step Chronocoulometry (Charge-Time Curves)

Potential-step chronocoulometry provides useful information on the mechanism of membrane permeabilization by ionophores, when applied to mercury-supported tBLMs. It consists in subjecting the electrochemical system under investigation to a potential step from an initial value *E_i_* to a final value *E_f_* and in recording the charge *Q*(*t*) that flows as a consequence of this step as a function of time. When applied to a mercury-supported DPTL/phospholipid tBLM in the absence of ionophores, the charge transient triggered by a potential step is characterized by an initial flow of purely capacitive charge, lasting less than one millisecond. Upon incorporating an ionophore in the tBLM, a potential step from an initial potential *E_i_* to a sufficiently negative final potential *E_f_* causes the ionophore to induce a cation flow into the tetraethyleneoxy (TEO) hydrophilic spacer. This is revealed by a negative charge that adds to the background charge recorded in the absence of channels. This negative charge is due to electrons that flow along the external circuit and accumulate on the metal surface, to maintain the electroneutrality of the whole electrified interface interposed between the bulk metal and the bulk aqueous solution. If only inorganic cations, such as K^+^ or Na^+^, are allowed to translocate across the lipid bilayer moiety of the tBLM via the ionophore, as is often the case, then the electron charge density σ*_M_* accumulating on the metal surface is practically equal in magnitude and opposite in sign to the cation charge density accumulating in the hydrophilic spacer. In fact, the only other charge density located within the electrified interface is that present in the diffuse layer adjacent to the tBLM, which is much smaller than the other two [53]. As a rule, a series of potential steps is carried out from a fixed initial potential *E_i_*, positive enough to repel K^+^ ions from the TEO spacer, to progressively more negative final potentials *E_f_*. Ultimately, an *E_f_* value is attained at which the hydrophilic spacer is completely saturated by the cations of the electrolyte. This gives rise to a charge transient with a well-defined sloping plateau, which adds to the background charge. In the presence of potassium ions, the height of the plateau, as estimated by extrapolating it to *t* = 0, ranges from −45 to −50 μC·cm^−2^ and corresponds to the charge associated to the maximum amount of potassium ions that can be accommodated in the TEO spacer, as verified for different ionophores, such as the ion carrier valinomycin, and the channel-forming peptides gramicidin [16,53], melittin, monazomycin [30], distinctin, alamethicin [54], trichogin GA IV [55], dermcidin [56] and syringomycin E [57]. This charge corresponds to two potassium ions per DPTL molecule, denoting an appreciable hydration of the spacer. Incidentally, tBLMs supported by Au [9] or Ag [8] do not allow ionic charge measurements carried out by stepping the applied potential to sufficiently negative final values. In fact, the resulting charge vs. time curves are roughly linear, with a relatively high and constant slope that is maintained for an indefinitely long time [8]. The constant current responsible for this linear increase in charge is ascribed to a slight water electrolysis with hydrogen formation. The high hydrogen overpotential of mercury avoids this inconvenience.

**Figure 6:**
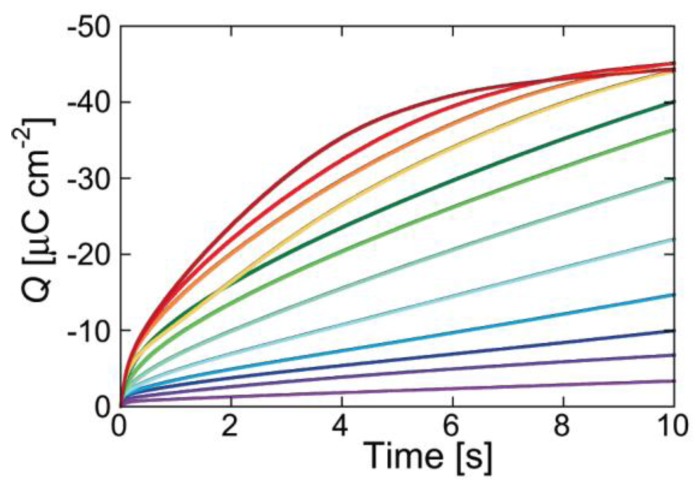
Charge vs. time curves following potential steps from a fixed initial potential of −0.200 V to progressively more negative final potentials, *E_f_*, varying from −0.525 to −0.925 V by −25 mV increments, at a DPTL/DPhPC tBLM incorporating gramicidin from its 0.1 μM solution in aqueous 0.1 M KCl. (Reprinted with permission from [16]. Copyright 2007, American Chemical Society).

The shape of the charge transients following *E_i_* → *E_f_* potential steps provides valuable information on membrane permeabilization by ionophores. Let us first consider the charge transients recorded at a DPTL/DPhPC tBLM incorporating gramicidin and shown in Figure 6 [16]. The charge increases with time first linearly and then, at longer times, less than linearly, turning the concavity toward the time axis. As *E_f_* is made progressively more negative, the charge transient becomes steeper, ultimately attaining a plateau whose extrapolation to *t* = 0 yields a charge density ranging from −45 to −50 μC·cm^−2^.

**Figure 7:**
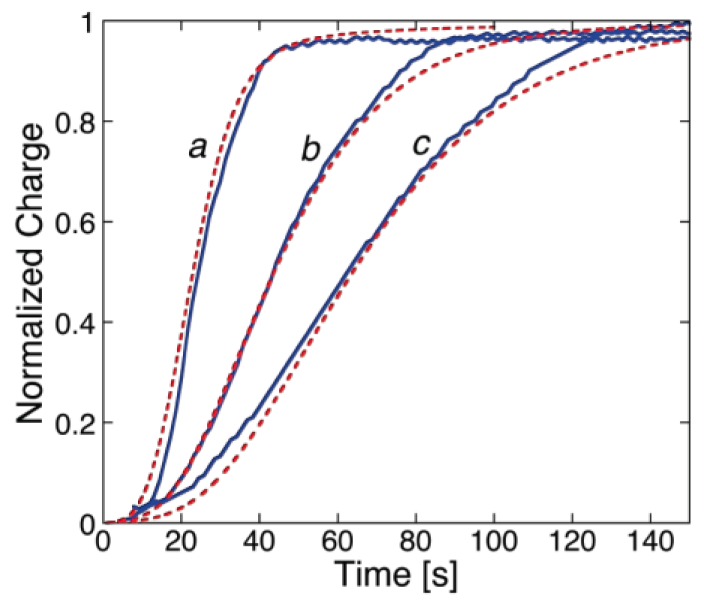
The solid blue curves are three successive charge vs. time curves at a DPTL/DPhPC tBLM following potential steps from the same initial value −0.20 V to the final values −1.05 V (a), −1.00 V (b) and −0.95 V (c) in aqueous 0.1 M KCl containing 0.14 μM melittin. The height of the plateau of the curves is normalized to unity. The corresponding dashed red curves were calculated using the parameters *θ*_0_ = 0.2, *n* = 2, and k*_N_k_R_*^2^ = 0.05 s^−3^ for all three curves; *p* was given the value 1 for curve (a), 0.8 for curve (b) and 0.6 for curve (c). (Reprinted with permission from [30]. Copyright 2007, American Chemical Society).

While ohmic channels start moving ions into the TEO spacer in the proximity of zero transmembrane potential *φ_m_*, voltage-gated channels show this behavior only at sufficiently negative *φ_m_* values. Moreover, their charge transients invariably exhibit a sigmoidal shape, as shown in Figure 7 for a DPTL/DPhPC tBLM incorporating melittin from its 0.14 μM solution in 0.1 M KCl [30].

This shape is semi-quantitatively interpreted on the basis of a model accounting for the formation of an ion channel by aggregation of monomeric units via a kinetic mechanism of nucleation and growth analogous to that already outlined for conventional BLMs. The fact that the sigmoidal shape typical of nucleation-and-growth kinetics is exhibited by current transients at BLMs and by charge transients at Hg-supported tBLMs is simply due to the replacement of one aqueous semi-infinite phase by a hydrophilic spacer in passing from BLMs to tBLMs. The ions moving across the lipid bilayer moiety of a tBLM are not free to diffuse in a semi-infinite aqueous medium on the *trans* side of the bilayer, as in the case of a BLM. Rather, they accumulate within the hydrophilic spacer moiety of the tBLM, spreading radially from the mouth of each newly formed ion channel, until they completely saturate the spacer. Thus, the current attains a maximum and then decays to zero, as soon as saturation is reached. Consequently, it is the charge vs. time curve that exhibits a sigmoidal shape, rather than the current-time curve, attaining a plateau that depends exclusively on the spaciousness of the hydrophilic spacer, irrespective of the number density of the ion channels. The model outlined in connection with potential step chronoamperometry is, therefore, modified by associating the nucleation and growth of ion channels to the concomitant radial diffusion of the translocating ions into the hydrophilic spacer [30].

The finite and well-defined speciousness of the TEO spacer allows the ionic charge entering it to be “dosed” as a function of the time elapsed from the instant of the potential step and of the final potential *E_f_*. Moreover, varying the rest time at *E_i_* between two consecutive *E_i_* → *E_f_* potential steps allows an estimate of the lifetime of the ion channel at the initial potential *E_i_*, just after its formation during the preceding negative potential step, as well as the rest time at *E_i_* required by the channel to expel the cations from the spacer into the aqueous solution. Thus, e.g., upon repeating a potential step that yields a sigmoidal charge transient at a DPTL/DOPC tBLM in an unbuffered solution of 0.1 M KCl and 4 μg/mL dermcidin-1L after a rest time of 30 s at *E_i_*, a much steeper charge transient of the same height is recorded, albeit lacking the sigmoidal shape [56]. This indicates that a rest time of 30 s at *E_i_* is not sufficient to dismantle the ion channels formed at *E_f_* as a consequence of the potential step. On the other hand, if the rest time at *E_i_* is of only 3 s, the charge step is as steep as in the previous case, albeit smaller. This proves that a time period of 3 s at *E_i_* is not sufficient to expel all the positive ions from the hydrophilic spacer.

**Figure 8:**
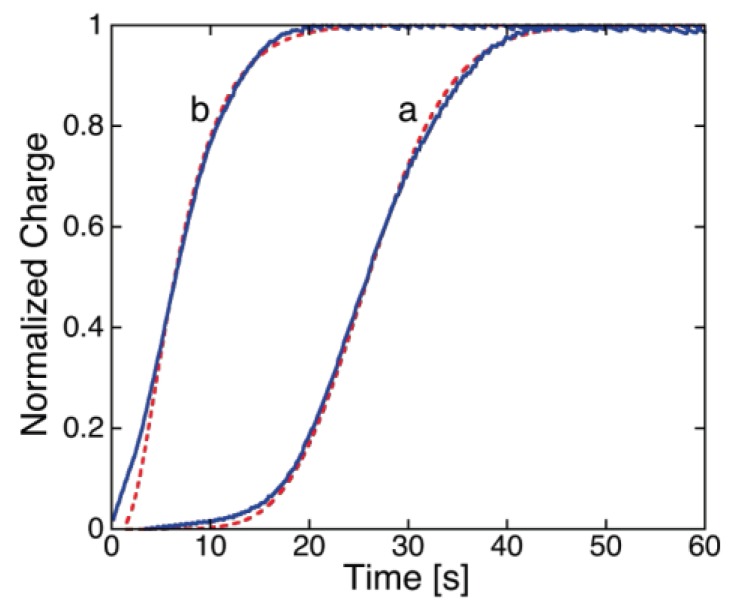
The solid curves are charge vs. time curves at a DPTL/DPhPC tBLM following potential steps from −0.20 to −1.05 V in aqueous 0.1 M KCl containing 0.14 μM melittin. Curve (a) was obtained after keeping the potential at −0.20 V for 15 min; curve (b) was obtained after keeping the potential at −1.05 V for 150 s, stepping it back to −0.20 V and carrying out the recorded potential jump to −1.05 V immediately after. The dashed curve (a) was calculated using the parameters *θ*_0_ = 0.2, *n* = 2, *k_h_*_,*N*_*v_h_*_,*R*_^2^ = 0.08 s^−3^, *k_N_k_R_*^2^ = 0.05 s^−3^ and *p* = 1; the dashed curve (b) was calculated using the parameters *θ*_0_ = 0.2, *n* = 2, *k_N_k_R_*^2^ = 40 s^−3^ and *p* = 1. (Reprinted with permission from [30]. Copyright 2007, American Chemical Society).

Several voltage-gated ion channels yield charge transients exhibiting the typical sigmoidal shape only after a relatively long time *t* from the instant of the potential step. In this case, the charge transient, after subtraction of the background charge, shows a “long foot” that may last several tens of seconds before the appearance of the sigmoidal curve. This is the case with monazomycin [30], distinctin and alamethicin [54]. The origin of the long foot is again to be ascribed to the disruption of flat clusters originally adsorbed on top of the tBLM and only indirectly affected by the applied potential; it can be quantitatively interpreted by an approach entirely analogous to that outlined for conventional BLMs [30]. The presence of a long foot confirms the existence of aggregates of peptide monomers on top of the tBLM at the initial potential *E_i_*. Its gradual development during the rest time at *E_i_*, immediately after forming a tBLM into a solution containing the peptide in monomeric form, measures the rate of peptide aggregation on top of a membrane. Thus, if a freshly formed DPTL/DPhPC tBLM is kept in the presence of 0.14 μM melittin for 15 min at *E_i_* before stepping the potential to a sufficiently negative value *E_f_*, the resulting charge transient shows a moderately long foot, which is absent if the rest time at *E_i_* is reduced to a minimum [30] (see Figure 8).

**Figure 9:**
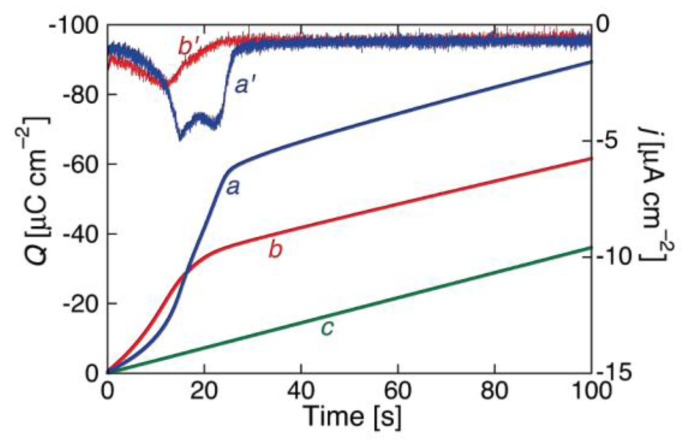
Charge transients following potential steps from *E_i_* = −0.30 V to two different final potentials *E_f_* at a DPTL/DOPS tBLM in a pH 3 solution of 0.1 M KCl and 0.8 μM SR-E. (a) Pristine potential step to *E_f_* = −0.90; (b) potential step to *E_f_* = −0.75 V recorded immediately after a potential step from −0.30 to −1.00 V and a rest time of 30 s at *E_i_*. The curves a′ and b′ are the corresponding current transients. Curve c is the charge transient from −0.30 V to −1.00 V in the absence of SR-E under otherwise identical conditions. (Reprinted and minimally adapted with permission from [57]. Copyright 2015, Elsevier).

A peculiar charge transient exhibiting two inflection points, namely two subsequent sigmoidal charge steps, is shown by the voltage-gated lipodepsipeptide syringomycin E (SR-E), upon stepping the potential from −0.30 to −0.90 V [57]. The two inflection points are hardly visible in the charge transient of curve *a* in Figure 9, although they are clearly apparent from the two peaks in the corresponding current transient of curve *a*′ in the same figure. Their origin is quite probably to be related to the existence of two types of ion channels differing in conductance by a factor of sixfold, as revealed by single-channel current measurements [58]. The channels of higher conductance (the large channels) are considered as clusters of the small ones exhibiting synchronous opening and closing. If a potential step from −0.30 to −1.00 V is immediately followed by a further potential step from −0.30 to −0.75 V, a sigmoidal charge transient with a single inflection point and the corresponding current transient with a single peak are obtained, as shown by curves *b* and *b*′ in Figure 9. The charge transient *b* has practically the same height as the first sigmoidal stage of the two-stage charge transient *a* for *E_f_* = −0.90 V, suggesting that the less negative final potential *E_f_* = −0.75 V allows the opening of only one of the two ion channels.

**Figure 10:**
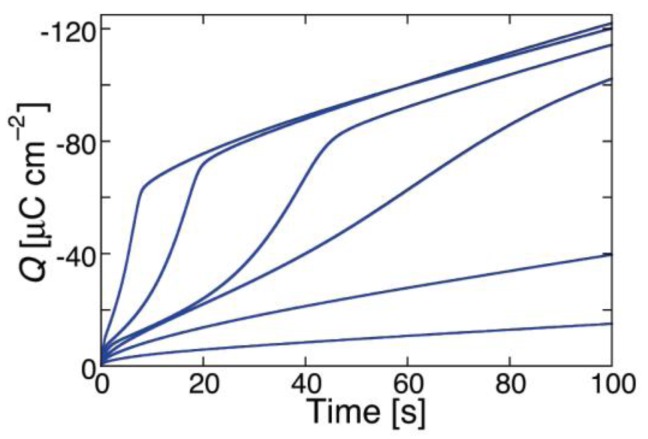
Charge transients at a DPTL/DOPC tBLM in a pH 6.8 buffer solution of 0.1 M KCl and 1 μg/mL SP25A, obtained by jumping from *E_i_* = −0.30 V to final potentials varying from −0.50 to −1.00 V by −100 mV increments. (Reprinted with permission from [18]. Copyright 2015, Elsevier).

While SR-E forms voltage-gated channels, syringopeptin 25A (SP25A), another lipodepsipeptide with a longer peptide chain produced by the Gram-negative bacterium *Pseudomonas syringae* pv. *syringae*, forms ohmic ion channels [18]. A remarkable difference with respect to the gramicidin ohmic channel, which is strictly cation-selective, is represented by the fact that SP25A has a poor ion selectivity and translocates both monovalent cations and anions. Under certain experimental conditions, this difference is revealed by an altogether different behavior of the charge transients at a Hg-supported tBLM. Thus, if the ion channel is already formed and in the open state at the initial potential *E_i_*, which corresponds to a positive transmembrane potential *φ_m_*, then during the rest time at *E_i_* in a KCl solution, Cl^−^ ions enter the TEO spacer in an amount depending of the magnitude of *φ_m_*. In this case, a sufficiently negative potential step yields a charge transient with a plateau appreciably higher than −50 μC·cm^−2^. This is due to the fact that the electrons flowing along the external circuit to ensure the electroneutrality of the whole electrified interface must compensate not only the positive charge of the K^+^ ions flowing into the hydrophilic spacer, but also the negative charge due to the concomitant outflow of the Cl^−^ ions that are present in the spacer at the initial potential *E_i_*. The charge transients induced by 1 μg/mL SP25A at a DPTL/DOPC tBLM in a pH 6.8 buffer solution of 0.1 M KCl attain a maximum height of about −60 μC·cm^−2^ and have a sigmoidal shape (see Figure 10).

However, a more attentive observation reveals that the lower portion of the charge transients exhibits a downward concavity that is maintained throughout the whole 100 s time window for *E_f_* ≥ −0.60 V and is confined within shorter time intervals as *E_f_* becomes progressively more negative. The presence of two stages can be explained, similarly to that observed with SR-E, by the presence of a large and a small single channel current, as recorded at BLMs by Dalla Serra et al. [17]. The large current has a lifetime of several seconds, whereas the small current is exactly one fourth of the large current and has an almost three orders of magnitude smaller lifetime. In view of the above behavior, the large current was tentatively ascribed to a large channel resulting from the tetrameric aggregation of small channels, leading to their synchronous opening and closing, in analogy with the hexameric clusters proposed for SR-E [58].

**Figure 11:**
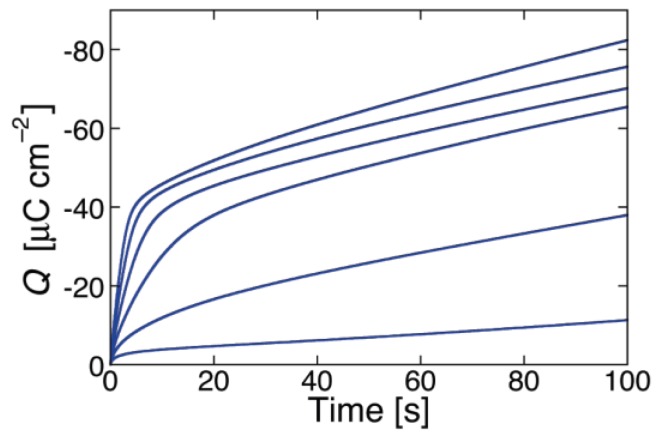
Charge transients at a DPTL/DOPC tBLM in a pH 6.8 buffer solution of 0.1 M KCl and 0.4 μg/mL SP25A, obtained by jumping from *E_i_* = −0.30 V to final potentials varying from −0.50 to −1.00 V by −100 mV increments. (Reprinted with permission from [18]. Copyright 2015, Elsevier).

The ascription of the two stages of the charge transient in Figure 10 to two different channels is supported by the charge transients in Figure 11, recorded at a SP25A concentration of 0.4 μg/mL under conditions otherwise identical with those in Figure 10. In this case, the charge transients do not show two distinct stages, pointing to an ion flow along a single type of ion channel, probably the small one. In fact, the lower peptide concentration may prevent a significant aggregation of the small channels into the large ones. The maximum charge attained in Figure 11 amounts to about 38 μC·cm^−2^, and is therefore less than that required to saturate the TEO spacer by the sole K^+^ ions. Most significantly, the charge transients in Figure 11 turn their concavity toward the time axis and do not exhibit a sigmoidal shape, differently from the one-stage charge transient induced by the voltage-gated SR-E channel at *E_f_* = −0.75 V in Figure 9, curves *b* and *b*′. This is a clear indication that the SP25A ion channel is already present at the initial potential *E_i_*, contrary to the SR-E one, which is only formed during the negative potential step by a kinetic mechanism of nucleation and growth.

At a DPTL/dioleoylphosphatidylserine (DOPS) tBLM, SP25A yields two-stage charge transients similar to those recorded at the DPTL/DOPC tBLM in Figure 10, at both pH 3 and 5.4 [59]. Their plateau attains a maximum height of −80 μC·cm^−2^ at pH 3, where the DOPS distal monolayer is slightly positively charged, and of −65 μC·cm^−2^ at pH 5.4, where it is substantially neutral [60]. Conversely, at pH 6.8, where DOPS is slightly negatively charged, the DPTL/DOPS tBLM yields one-stage, concave downward charge transients attaining a maximum height of about −45 μC·cm^−2^, similarly to those provided by gramicidin [16]. The fact that the charge transients are concave downward indicates that the ion channel is already formed at the initial potential *E_i_*; moreover, the fact that their maximum height corresponds to spacer saturation by K^+^ ions indicates that the ion channel is unable to translocate Cl^−^ ions. In other words, it is in the closed state at positive transmembrane potentials. This result can be rationalized by assuming that the SP25A channel opens whenever the positive transmembrane potential succeeds in pushing the positively charged octadepsipeptide moiety of SP25A slightly out of the membrane, as proposed by Dalla Serra et al. [17]. This occurs when the distal monolayer is practically uncharged, namely at a DPTL/DOPC tBLM and at a DPTL/DOPS tBLM at pH 5.4 and, even more so, when the distal monolayer is positively changed, namely at a DPTL/DOPS tBLM at pH 3. Conversely, when the distal monolayer is negatively charged, as in the case of the DPTL/DOPS tBLM at pH 6.8, the negative charge may prevent the octadepsipeptide moieties at the SP25A channel mouth from being pushed backwards by a positive transmembrane potential, thus maintaining the channel closed state.

### 3.2. What Can Be Learnt from Electrochemical Impedance Spectroscopy (EIS)

Electrochemical impedance spectroscopy applies an AC voltage of given frequency to the system under study and measures the resulting current that flows with the same frequency. Both the amplitude of the AC current and its phase shift with respect to the AC voltage are measured. The frequency is normally varied gradually from 10^−3^ to 10^5^ Hz. A tBLM can be regarded as a series of slabs with different dielectric properties. When ions flow across each slab following a perturbing AC voltage, they give rise to an ionic current *I*_ion_, in phase with the AC voltage. Ions also accumulate at the boundary between contiguous dielectric slabs; under the AC voltage, the extent of this accumulation varies with time, giving rise to a capacitive current *I_c_*, in quadrature with the AC voltage. The total current is, therefore, given by the vector sum of the ionic current and of the capacitive current. In this respect, each dielectric slab can be simulated by a resistance, accounting for the ionic current, in parallel with a capacitance, accounting for the capacitive current. This parallel combination of a resistance *R* and a capacitance *C* is referred to as an “*RC* mesh”. Accordingly, the impedance spectrum of a tBLM can be simulated by a series of *RC* meshes.

Application of an AC voltage of amplitude *v* and frequency *f* to a pure resistor of resistance *R* yields a current of equal frequency *f* and amplitude *v/R*, in phase with the voltage. Conversely, application of the AC voltage to a pure capacitor of capacitance *C* yields a current of frequency *f* and amplitude 2*πfC*, out of phase by –*π*/2 with respect to the voltage, i.e., “in quadrature” with it. This state of affairs can be expressed by stating that the admittance *Y* of a resistance element equals 1/*R*, while that of a capacitance element equals −*iωC*, where *ω* = 2*πf* is the angular frequency and *i* is the imaginary unit. More generally, in an equivalent circuit consisting of resistances and capacitances, *Y* is a complex quantity, and the impedance *Z* is equal to 1/*Y*, by definition. Denoting the in-phase and quadrature components of the impedance by *Z′* and *Z″*, respectively, a single *RC* mesh yields a semicircle of diameter *R* and center of coordinates (*R*/2,0) when displayed on a *Z″* vs. *Z′* plot, called Nyquist plot [4]. Moreover, the angular frequency *ω* at the maximum of the semicircle equals the reciprocal of the time constant, *RC*, of the mesh.

In the presence of a series of *RC* meshes, their time constants may be close enough to cause the corresponding semicircles to overlap partially. In this case, if the *RC* mesh of highest time constant has also the highest resistance, *R*_1_, as is often the case, then the Nyquist plot of the whole impedance spectrum exhibits a single well-formed semicircle, *R*_1_ in diameter. The semicircles of the remaining *RC* meshes are compressed in a very narrow area close to the origin of the *Z″* vs. *Z′* plot, and can be visualized only by enlarging this area. To better visualize all semicircles, we have found it convenient to represent impedance spectra on a *ωZ′* vs. –*ωZ″* plot, briefly referred to as an “M plot”, since *ωZ′* and *ωZ″* are the components of the modulus function *M* [4]. Even in this plot, a single *RC* mesh yields a semicircle, with diameter *C*^−1^ and center of coordinates (1/(2*C*),0). Moreover, *ω* at the maximum of the semicircle is again equal to the reciprocal of the time constant *RC* of the mesh. While *ω* decreases along the positive direction of the abscissa on a *Z″* vs. *Z′* plot, it increases on an M plot. Therefore, for a series of *RC* meshes, the last semicircle on the M plot is characterized by the lowest time constant. This is, unavoidably, the semicircle simulating the solution that baths the self-assembled film, due to its very low capacitance. As a rule, for a series of *RC* meshes, an M plot determines a more even distribution of their time constants along the abscissa than the corresponding Nyquist plot.

**Figure 12:**
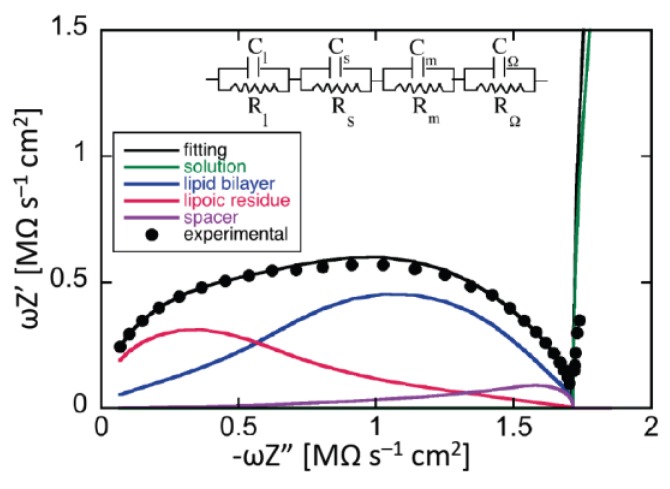
Plot of *ωZ*′ (black circles) against –*ωZ*″ for a mercury-supported DPTL/DPhPC bilayer immersed in aqueous 0.1 M KCl at −0.41 V vs. SCE. The black curve is the best fit of the impedance spectrum by the equivalent circuit shown in the inset, with *R_l_* = 17 MΩ·cm^2^, *C*_l_ = 1.6 μF·cm^−2^, *R_s_* = 0.18 MΩ·cm^2^, *C_s_* = 5.5 μF·cm^−2^, *R_m_* = 4.2 MΩ·cm^2^, *C*_m_ = 1.1 μF·cm^−2^, *R*_Ω_ = 3.6 Ω·cm^2^, *C*_Ω_ = 27 nF·cm^−2^. The colored curves are contributions to *ωZ*′ from the single slabs.

A tBLM can be simulated by a maximum of four different dielectric slabs, consisting of the lipoic acid moiety directly tethered to the mercury surface, the hydrophilic spacer moiety, the lipid bilayer moiety (cf. Figure 5), and a thin shall of aqueous solution adjacent to the lipid bilayer surface. Consequently, the tBLM can be simulated by the “equivalent circuit” shown in the inset of Figure 12 and consisting of four meshes in series, (*R_l_C_l_*)(*R_s_C_s_*)(*R_m_C_m_*)(*R*_Ω_C_Ω_); here, the subscripts *l*, *s*, *m* and Ω denote the lipoic acid moiety, the spacer moiety, the lipid bilayer moiety and the aqueous solution. In the absence of exogeneous species incorporated in the lipid bilayer moiety, *C_l_*, *C_s_* and *C_m_* amount to 2–5, 3–7 and 0.8–1 μF·cm^−2^, respectively, whereas the corresponding resistances *R_l_*, *R_s_* and *R_m_* amount to 5–20, 0.05–0.2 and 2–5 MΩ·cm^2^ [61]. Moreover, at a tBLM in aqueous solution of 0.1 M KCl, *C*_Ω_ and *R*_Ω_ are about equal to 25 nF·cm^−2^ and 4 Ω·cm^2^. Hence, the time constant of the lipoic acid moiety is of the order of 10 s, those of the spacer and lipid bilayer moieties are comparable and of the order of 1 s, and that of the aqueous solution is of the order of 10^−7^ s. At −0.41 V vs. SCE, the semicircles that we meet when proceeding along the positive direction of the horizontal –*ωZ″* axis of the M plot (i.e., toward increasing frequencies) are ascribable, in the order, to the lipoic acid moiety, the lipid bilayer, the spacer and the aqueous solution, as shown in Figure 12. Following how the different RC meshes vary with the applied potential allows one to assign them to the appropriate dielectric slab of the tBLM [61].

The first three semicircles are notably distorted due to their appreciable overlapping. This is clearly indicated by the plots of the contributions to *ωZ*′ from the single slabs against –*ωZ*″, which serve to show how each semicircle is affected by partial overlapping with the neighboring ones. In fact, each calculated contribution is represented by a semicircle that is distorted the more, the higher the overlapping. The fourth semicircle is not distorted because of its much lower time constant; moreover, it has a very large diameter due to the very low capacitance of the solution, and only a small portion of it is visible at the highest frequencies (≤10^5^ Hz) covered by the EIS instrumentation.

**Figure 13:**
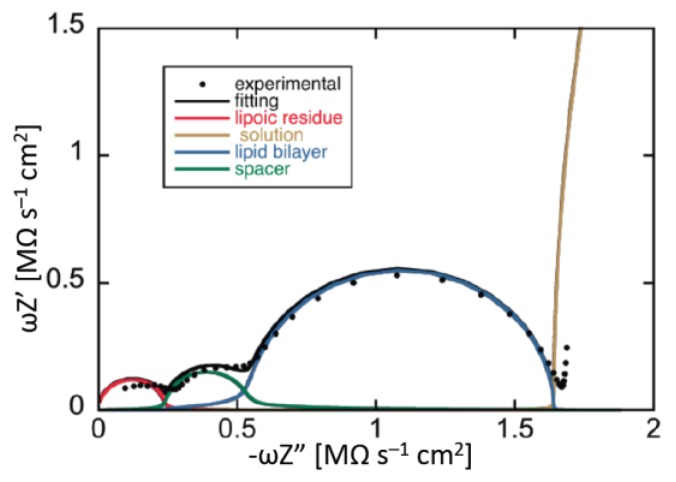
Plot of *ωZ*′ (solid circles) against −*ωZ*″ for a mercury-supported DPTL/DPhPC bilayer incorporating valinomicin from its 0.15 μM solution in aqueous 0.1 M KCl at −0.41 V vs. saturated calomel electrode (SCE). The solid black curve is the best fit of the impedance spectrum by the equivalent circuit shown in the inset of Figure 12, with *R_l_* = 2.8 MΩ·cm^2^, *C_l_* = 4.2 μF·cm^−2^, *R_s_* = 48 kΩ·cm^2^, *C_s_* = 3.3 μF·cm^−2^, *R_m_* = 3.4 kΩ·cm^2^, *C_m_* = 0.9 μF·cm^−2^, *R*_Ω_ = 3.7 Ω·cm^2^, *C*_Ω_ = 43 nF·cm^−2^. The colored curves are contributions to *ωZ*′ from the single slabs. (Adapted with permission from [61]. Copyright 2005 American Chemical Society).

Reconstituting an ion channel such as gramicidin or melittin, or an ion carrier such as valinomycin, into the lipid bilayer moiety of a tBLM in contact with aqueous 0.1 M KCl changes the capacitances of the various slabs of the tBLM only slightly. Conversely, it decreases the resistance of the lipoic acid moiety by about one order of magnitude, that of the spacer by about two orders of magnitude and that of the lipid bilayer by about three orders of magnitude. The *RC* mesh of the aqueous solution does not depend on the architecture of the tBLM; hence, the corresponding semicircle is usually excluded almost completely from an M plot. The largest effect of an ion channel or carrier is clearly produced on the lipid bilayer moiety, where these exogenous species penetrate moving ions across it. The different extent of this effect on the three different dielectric slabs decreases the overlapping between the corresponding semicircles, as shown in the M plot of Figure 13 at a tBLM incorporating valinomycin [61], where the three semicircles are clearly distinguishable. Note that the decrease in resistance being higher in the lipid bilayer than in the spacer causes the time constant of the former to become lower than that of the latter, inverting the position of the corresponding semicircles along the –*ωZ*″ axis (cf. Figure 12 and Figure 13). Similar semicircles in the M plot are also obtained by incorporating gramicidin [16] or melittin [62] in the tBLM.

**Figure 14:**
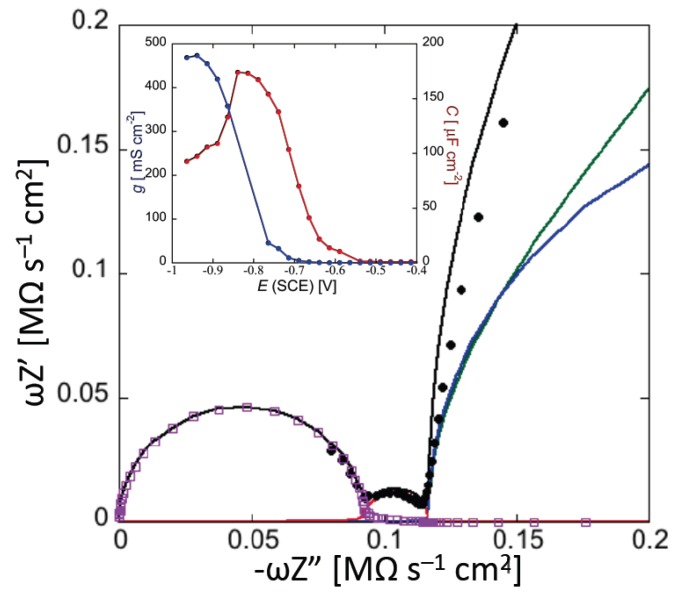
Plot of *ωZ*′ (solid circles) against −*ωZ*″ at a Hg-supported DPTL/DPhPC tBLM incorporating gramicidin from its 0.1 μM solution in aqueous 0.1 KCl at −0.675 V vs. SCE. The solid black curve is the best fit of this plot by an equivalent circuit consisting of a series of four *RC* meshes. The purple, red, blue and green curves are calculated contributions to *ωZ*′ from the spacer moiety, the “inner layer” moiety, the lipid bilayer moiety and the aqueous solution adjacent to the BLM. The inset shows plots of conductance *g* (blue curve) and capacitance *C* (red curve) of the inner layer against *E* at the same tBLM. (Reproduced from [53] with permission from the Royal Society of Chemistry).

At applied potentials negative enough to allow potassium ions to accumulate in the TEO spacer to an appreciable extent, the *R_l_C_l_* mesh ascribed to the lipoic acid moiety is no longer detectable; it is replaced by an *RC* mesh characterized a by a higher capacitance, and hence by a smaller diameter of the corresponding semicircle in the M plot. This *RC* mesh is clearly visible in the impedance spectra of tBLMs incorporating either valinomycin or gramicidin. Figure 14 shows the M plot for a tBLM incorporating gramicidin from its 0.1 μM solution in aqueous 0.1 M KCl at −0.675 V vs. SCE (solid black circles), together with the corresponding fit by an equivalent circuit consisting of four *RC* meshes in series (solid black curve) [53]. The calculated contribution to *ωZ′* from each single *RC* mesh is also plotted against the overall –*ωZ″* quantity. It is evident that the overlapping of the semicircle of smallest diameter and highest capacitance with the neighboring semicircles is modest, thus allowing an accurate estimate of its capacitance. The potential dependence of the differential capacitance *C* and conductance *g* = 1/*R* of the slab of highest capacitance is shown in the inset of Figure 14. The capacitance increases in parallel with the charge of potassium ions in the TEO spacer, attaining a maximum value of about 200 μF·cm^−2^. This capacitance is to be attributed to the region of the lipoic acid residues, which is about equal to 5 μF·cm^−2^ in the absence of ions in the spacer [61] and increases in a roughly linear way with an increase in the cation charge in the spacer. This is because this region (or, more precisely, the part of it closest to the mercury surface) becomes gradually sandwiched between this cation charge and an almost equal and opposite charge on the metal surface, due to an excess of conduction electrons.

The ionic charge density in the hydrophilic spacer is practically equal in magnitude and opposite in sign to the charge density on the metal surface, to ensure the electroneutrality of the whole mercury/(aqueous solution) electrified interface. Water molecules and partly desolvated K^+^ ions are intercalated between the lipoic acid residues anchored to the mercury surface. We can, therefore, envisage an “inner layer” analogous to that considered in connection with bare mercury in the presence of ionic specific adsorption. The capacitance, *C*_il_, of this inner layer can be approximately expressed by the equation:

(15) $$c_{il}=\left[5+\frac{F\Gamma }{45}195\right]\mu F\;{\text{cm}}^{-2}$$

It accounts for the progressive evolution from its initial value, *C_l_* ≅ 5 μF·cm^−2^, in the absence of ions in the spacer [61], to its maximum value of 200 μF·cm^−2^, as the surface charge density, *FГ*, of monovalent cations in the TEO spacer increases, tending to its maximum value of +45 μC·cm^−2^.

### 3.3. The Absolute Potential Difference ∆φ across the Mercury/(Aqueous Solution) Interface

An estimate of the extra-thermodynamic absolute potential difference across the overall interphase between the bulk metal and the bulk aqueous phase, ∆*φ*, is of importance to compare the behavior of a metal-supported biomimetic membrane with that of a BLM. This comparison can hardly be made at a solid metal support, such as Au or Ag, because of its critical dependence upon the mono- or polycrystalline structure, surface defects, potential-dependent reconstruction phenomena, etc. Conversely, a sufficiently accurate estimate of ∆*φ* with respect to a conventional reference electrode can be made at a hanging mercury drop electrode (HMDE), thanks to its liquid state and perfectly smooth and reproducible surface. To this end, let us consider a DOPC monolayer self-assembled on a mercury drop immersed in an aqueous electrolyte, with its hydrocarbon tails directed toward the hydrophobic mercury surface and the polar heads turned toward the aqueous phase. ∆*φ* is expressed by the equation [63]:

(16) $$\Delta \phi =\frac{\sigma_{M}}{C}+\chi +\psi \left(c,\sigma_{M}\right)\;\text{with}\;\chi =Nm/\left(\varepsilon_{0}\varepsilon_{\gamma }\right)$$

Here, *σ_M_* is the charge density on the metal, *C* is the differential capacitance of the monolayer, *χ* is the surface dipole potential due to the lipid polar heads, *m* is the dipole moment normal component of the latter, *N* is the number density of the lipid molecules, *ε_γ_* is the dielectric constant of the polar head region and *ε*_0_ is the permittivity of free space. Moreover, *ψ*(*c*,*σ_M_*) is the potential difference across the diffuse layer, regarded as a function of the electrolyte concentration *c* and of *σ_M_* according to the Gouy-Chapman theory. The capacitance *C* can be regarded as a series combination of the capacitance, *C*_ht_, of the hydrocarbon tail region and of that, *C*_ph_, of the polar head region. Since *C*_ph_ is much greater than *C*_ht_, *C* practically coincides with *C*_ht_. On the other hand, the surface dipole potential of the hydrocarbon tail region is negligible, because of the very small difference between the electronegativities of the carbon and hydrogen atoms. This is not the case for the surface dipole potential *χ* of the polar head region, which must be included in Equation (16).

While *C* and *σ_M_* are thermodynamically significant quantities, in that their measurement does not require modelistic assumptions, *χ* is not, and can only be estimated on the basis of extra-thermodynamic considerations. This goal can be achieved by gradually expanding the lipid-coated mercury drop inside the aqueous electrolyte and by measuring the charge density *σ_M_* that accompanies this expansion [64]. The amount of lipid material on the drop surface remains constant during the drop expansion, while the self-assembled molecules undergo a progressive tilt without incorporating water molecules. Denoting by *θ* the tilt angle of the self-assembled lipid molecules with respect to the monolayer normal, the monolayer thickness, *d*(*θ*), at a given stage of the expansion is given by *d* cos*θ*. The quantities whose dependence upon *θ* is not explicitly expressed will be referred to the initial unexpanded drop. The expansion of the drop area, *A*(*θ*), does not alter the monolayer volume, *A*(*θ*) × *d*(*θ*) = *A* × *d*; it follows that *A*(*θ*) is just equal to *A*/cos*θ*, allowing cos*θ* to be calculated from the *A*/*A*(*θ*) ratio. Moreover, the constancy of the number of monolayer molecules during the drop expansion causes their number density *N* to decrease as *N*(*θ*) = *NA*/*A*(*θ*) = *N* cos*θ* and *m* to decrease as *m*(*θ*) = *m* cos*θ*. Finally, the drop expansion causes the capacitance to increase as *C*(*θ*) = *ε*_0_*ε_γ_*/*d*(*θ*) = *C*/cos*θ*. In view of Equation (16), monitoring the charge density *σ_M_*(*θ*) during the gradual expansion at constant applied potential, *E* = ∆*φ* + constant, allows the *χ* value to be estimated at +145 ± 10 mV from the opposite of the slope of the linear plot of {*σ_M_*(*θ*) cos*θ*/*C* + *ψ*[*c*,*σ_M_*(*θ*)]} against cos^2^*θ* [64]. Values estimated by other extrathermodynamic procedures at conventional BLMs range from +150 to +250 mV.

The plot of the differential capacitance *C* at DOPC-coated mercury against *E* in aqueous solution of 0.1 M KCl shows a flat minimum over a potential range of about 600 mV straddling −0.450 V vs. SCE, where *C* is practically equal to 1.8 μF·cm^−2^ and the dipole moment *m* does not change orientation [56]. Hence, any applied potential over this range can be indifferently chosen to compare the potential scale relative to the saturated calomel electrode (SCE) with the absolute potential scale expressed by Equation (16). Thus, e.g., at −0.45 V vs. SCE, *σ_M_* equals −0.75 μC·cm^−2^; inserting this value into Equation (16), together with *C* = 1.8 μF·cm^−2^ and *χ* = +0.200 V, one obtains an approximate ∆*φ* value of −0.220 ± 0.050 V, which is more positive than the corresponding *E* value by 0.230 V [63]. Therefore, the absolute potential difference ∆*φ* across the Hg/(aqueous solution) interface at any given applied potential can be roughly estimated by adding +0.230 V to the applied potential measured vs. SCE, irrespective of the interfacial region interposed between bulk mercury and the bulk aqueous phase. It should be noted that the ∆*φ* ≈ (*E* vs. SCE + 0.230 V) value differs from the “true” absolute potential difference across the Hg/(aqueous solution) interface by the surface dipole potential due to the electron spillover, whose positive value in vacuum, *χ*_e_^Hg^, varies by an amount *δχ*_e_^Hg^ due to the molecules in direct contact with the mercury surface. The quantity *δχ*_e_^Hg^ depends on whether the molecules in contact with the mercury surface are water molecules, the hydrocarbon tails of a lipid self-assembled monolayer (SAM) or the sulfhydryl groups of a thiol monolayer, where the surface dipole potential of the Hg–S bond should also be accounted for [65]. It is upon ignoring the (*χ*_e_^Hg^ + *δχ*_e_^Hg^) contribution that a direct comparison between ∆*φ* values estimated at bare, SAM-coated and thiol-coated mercury can be reasonably made.

### 3.4. What Can Be Learnt from Cyclic Voltammetry Measurements

When applied to a Hg-supported tBLM in the absence of channel-forming peptides, a negative-going potential scan gives rise to a roughly constant negative capacitive current, due to a gradual accumulation of electrons on the metal surface and to a parallel accumulation of positive ions in the diffuse layer adjacent to the tBLM. In fact, the current density *j* is given by d*Q*/d*t* = (d*Q*/d*E*) (d*E*/d*t*) = *Cv*, where *Q* is the capacitive charge density, *C* is the differential capacitance and *v* is the potential scan rate. The current also includes a resistive contribution due to the non-infinite resistance of the lipid bilayer moiety, which imparts a slight tilt to the current trace. The reverse positive-going potential scan yields a current with the same resistive contribution but an opposite capacitive contribution with respect to the zero-current axis. This results in a trace whose shape is similar to that of a parallelogram.

The cyclic voltammograms (CVs) at mercury-supported tBLMs are quite different from the *I-V* curves recorded at conventional BLMs incorporating the same peptides. In the presence of an ion channel, the negative-going potential scan of a cyclic voltammetry curve yields a negative current peak that adds to the background current and is caused by the monovalent cation inflow into the hydrophilic spacer. The negative current starts increasing as soon as the potential difference across the lipid bilayer moiety of the tBLM (i.e., the transmembrane potential *φ_m_*) becomes slightly negative in the case of ohmic ion channels, but only at appreciably negative *φ_m_* values in the case of voltage-gated ion channels. Ohmic channels show both a positive and a negative current peak, which are roughly centrosymmetric with respect to the midpoint potential, *E*_1/2_, between the two peaks. The positive current peak is due to the cation outflow from the spacer along the ion channel. In the case of K^+^ inflow into the TEO spacer, integration of the current peak over time yields a charge density ranging from −45 to −50 μC·cm^−2^, practically equal and opposite to the K^+^ charge density saturating the spacer, at least at potential scan rates ≤50 mV/s.

**Figure 15:**
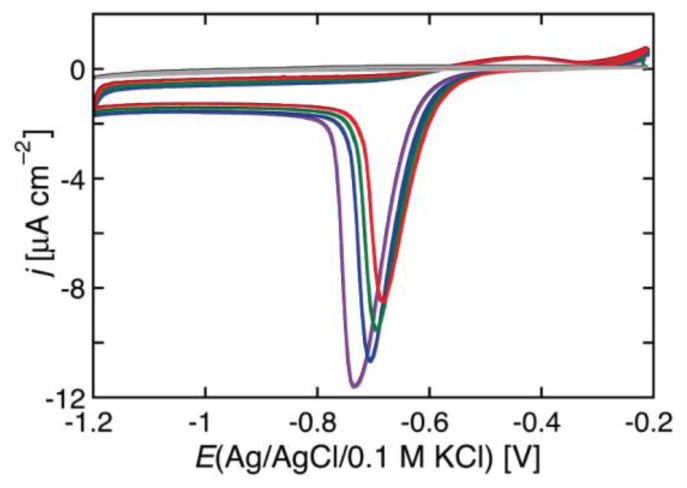
Cyclic voltammograms (CVs) at a DPTL/DOPC tBLM in a pH 3 solution of 0.1 M KCl, 1 × 10^−3^ M HCl and 0.8 μg/mL melittin, recorded between −0.20 and −1.20 V at a scan rate of 50 mV/s. Pristine CV (purple curve) and three further CVs (blue, green and red curves) after a few potential cycles. The grey curve is the CV in the absence of melittin. (Reprinted from [19]. Copyright 2016, with permission from Elsevier).

On the other hand, voltage-gated ion channels show only a small positive current hump, which is mainly induced by nonspecific ion leakage out of the spacer across the lipid bilayer. Repeated voltage cycles cause a gradual decrease of the negative current peak, since the slow nonspecific cation outflow at positive transmembrane potentials is unable to completely eject from the spacer the cation charge accumulated along the preceding negative current peak, as shown by melittin incorporated in a DPTL/DOPC tBLM [19] (see Figure 15). A steady state is ultimately attained when the cation charge expelled from the spacer during the positive current hump equals that required to saturate the spacer during the negative current peak. This cation charge is appreciably less than that corresponding to spacer saturation, and the charge under the negative current peak is practically equal and opposite to it.

As a rule, a non-physiological transmembrane potential *φ_m_* ranging from −300 to −350 mV is required to incorporate a voltage-gated channel at a freshly prepared tBLM, thus eliciting a negative current peak. However, after this initial incorporation, voltage-gated channels, such as melittin and the peptaibols alamethicin and trichogin GA IV [55], permeabilize the lipid bilayer stably at transmembrane potentials ranging from −80 to −90 mV. These are physiological transmembrane potentials, although their relatively high values are indicative of a voltage-gated behavior.

Cyclic voltammetry curves are not controlled by diffusion of the permeating ions in the aqueous solution toward or away from the tBLM surface. Rather, they are controlled by the rate at which the permeating ions overcome the potential energy barrier consisting of the lipid bilayer interposed between the aqueous solution and the hydrophilic spacer [66]. In other words, the concentration of a permeating ion in direct contact with the external surface of the tBLM does not change to a detectable extent with respect to its bulk value during the functional activity of an ion channel. Hence, cyclic voltammetry provides useful information on the kinetics of inflow and outflow of permeating ions, by also varying the scan rate. In particular, it allows a distinction to be made between ohmic and voltage-gated ion channels.

The midpoint potential, *E*_1/2_, at a tBLM incorporating an ohmic ion channel marks the dynamic equilibrium between the ions flowing into the spacer and those flowing out of it and, hence, it is equivalent to the zero transmembrane potential at a symmetric BLM interposed between two identical aqueous solutions. Even though the volume concentration of the ions at the inner mouth of the channel (i.e., in the spacer) does not necessarily coincide with that at its outer mouth (i.e., in the bathing aqueous solution) at *E*_1/2_, this potential can be regarded as an approximate measure of the zero potential difference across the lipid bilayer moiety of the tBLM, namely the zero transmembrane potential *φ_m_*. It is noteworthy that altogether different ohmic channels, such as the neutral dimeric gramicidin channel [66] and the positively charged polymeric SP25A channel [18], yield practically the same midpoint potential *E*_1/2_ and exhibit the same *E*_1/2_ dependence upon pH, once incorporated in the DPTL/DOPC tBLM. The same behavior is also shown by the melittin channel, under the particular experimental conditions where it behaves like an ionic channel [19]. For all the above ohmic channels, *E*_1/2_ shifts towards less negative potentials with decreasing pH, passing from ~ −0.65 V vs. Ag/AgCl(0.1 M KCl) at pH 6.8 to ~ −0.55 V at pH 5.4 and to ~ −0.45 V at pH 3. The fact that ohmic ion channels with quite different charge and structure show the same *E*_1/2_ dependence upon pH at the DPTL/DOPC tBLM is a strong indication that it stems from the pH sensitive part of the tBLM exposed to the aqueous solution, namely the ionizable groups of the DOPC polar heads that surround the channel mouth.

In this connection, it should be noted that the DOPC distal monolayer is characterized by an arrangement of the (H_3_C)_3_N^+^–CH_2_–CH_2_–OPO_3_^−^ zwitterion coplanar to the monolayer, due to electrostatic interactions between trimethylammonium and phosphate groups of adjacent lipid molecules, with a resulting stabilization of the unprotonated form of the latter group [60]. Due to the exposure of the phosphate group to the aqueous solution, its intrinsic pK_a_ is about equal to 0.8, and hence its protonation starts to become appreciable around pH 2. However, if the (H_3_C)_3_N^+^–CH_2_–CH_2_–OPO_3_^−^ dipoles of the DOPC molecules adjacent to the mouth of a gramicidin channel are dragged closer to the hydrocarbon tail region, they will tilt with respect to the bilayer plane, with the phosphate group (linked to the glycerol backbone) being in a more embedded position with respect to the corresponding trimethylammonium group. The decreased polarizability of the local environment surrounding the phosphate group will tend to favor its protonation more than if it were exposed to the aqueous phase, causing a progressive decrease in its negative charge with decreasing pH from 6.8 to 3. At constant applied potential *E*, the resulting decrease in the negative potential difference across the polar head region is compensated for by an increasing negative potential difference across the hydrocarbon tail region (i.e., the transmembrane potential *φ_m_*), causing an increase in the K^+^ inflow into the spacer relative to its outflow. Therefore, the electric potential at which the K^+^ outflow matches its inflow (i.e., the midpoint potential *E*_1/2_) is attained at less negative potentials with decreasing pH.

The experimental cyclic voltammetry behavior of the gramicidin ion channel can be interpreted semiquantitatively on the basis of the above considerations by having recourse to the following approximate model [19,66]. The mercury-supported DPTL/DOPC tBLM can be regarded as consisting of four consecutive dielectric slabs: (i) the region of the lipoic acid residues; (ii) the tetraethyleneoxy hydrophilic spacer; (iii*)* the lipid bilayer moiety; (iv) the polar head region. The potential difference across each slab equals the ratio of the charge density at its inner boundary (i.e., that turned toward the metal) to the corresponding capacitance. This charge density is clearly equal to the sum of all charge densities located between this boundary and the bulk metal. The charge density at the outer boundary of the given slab is necessarily equal in magnitude and opposite in sign to that at its inner boundary in view of the electroneutrality of the whole metal/water interface, since it is the sum of all charge densities enclosed between the outer boundary and the bulk aqueous phase. The negative charge density due to the disulfidated groups of the lipoic acid residues attached to the mercury surface, even if partially or totally transferred to the metal, is counterbalanced to a large extent by a defect of conduction electrons on the metal surface; the sum of these two charges, denoted by *q_M_*, is that experienced by the diffuse layer ions when no ion channels are present, and amounts to no more than a few μC·cm^−2^ over the potential range of interest [53,67]. The charge density of the monovalent cations entering the hydrophilic spacer by moving along the ion channel will be denoted by *FГ*, where *Г* is the cation surface concentration; as a first approximation, it will be regarded as mainly located on the metal side of the TEO spacer [53]. An ionic charge density *σ_i_* will be ascribed to any ionizable polar-head groups deeply embedded in the polar head region and located in the immediate vicinity of the hydrocarbon tails. With the above assumptions, the absolute potential difference ∆*φ* across the whole mercury/water interface is given by:

(17) $$\Delta \phi =\frac{q_{M}}{C_{il}}+\left(\frac{q_{M}+F\Gamma }{C_{sp}}+\chi_{sp}\right)+\frac{q_{M}+F\Gamma }{C_{m}}+\frac{\left(q_{M}+F\Gamma +\sigma_{i}\right)}{C_{ph}}$$

Here, *χ*_sp_ is the surface dipole potential of the TEO spacer, which contributes to the potential difference across this dielectric slab; *C*_il_, *C*_sp_, *C_m_* and *C*_ph_ are the differential capacitances of the inner-layer region (formed by the lipoic acid residues), the spacer, the lipid bilayer moiety and the polar heads, respectively. The linear dependence of *C*_il_ upon *Г* is expressed by Equation (15).

Upon extracting *q_M_* from Equation (17) as a function of ∆*φ* and substituting the resulting expression into the equation, *φ_m_* = (*q_M_* + *FГ*)/*C_m_*, for the transmembrane potential, after straightforward algebraic calculations we obtain:

(18) $$\phi_{m}\equiv \frac{q_{M}+F\Gamma }{C_{m}}=\frac{\Delta \phi +F\Gamma /C_{il}-\chi_{sp}-\sigma_{i}/C_{ph}}{C_{m}\left(C_{il}^{–1}+C_{sp}^{–1}+C_{m}^{–1}+C_{ph}^{–1}\right)}≅\Delta \phi +F\Gamma /C_{il}–\chi_{sp}-\sigma_{i}/C_{ph}$$

The last member of this equation is justified by the fact that the differential capacitance *C_m_* of the lipid bilayer moiety is always appreciably smaller that the other capacitances, such that the reciprocals of these latter can be disregarded with respect to *C_m_*^−1^ as a good approximation.

The current density *j* is obtained from the absolute rate theory of ion transport across membranes [47], as applied to the ion translocation across the potential energy barrier located in the lipid bilayer moiety of the tBLM. It is given by the sum, *j* = *j*^+^ + *j*^−^, of the contributions from the cation and anion flows:

(19) $$j^{+}=Fk_{t}^{+}\left[c_{sp}^{+}\;\text{exp}\left(\frac{\left(1-\alpha \right)F\phi_{m}}{RT}\right)-c_{0}\;\text{exp}\left(-\frac{\alpha F\phi_{m}}{RT}\right)\left(1-\frac{F\left|\Gamma \right|}{45}\right)\right]$$

(20) $$j^{-}=-Fk_{t}^{-}\left[c_{sp}^{-}\;\text{exp}\left(-\frac{\left(1-\alpha \right)F\phi_{m}}{RT}\right)-c_{0}\;\text{exp}\left(\frac{\alpha F\phi_{m}}{RT}\right)\left(1-\frac{F\left|\Gamma \right|}{45}\right)\right]$$

In this equation, *k^+^_t_* and *k^−^_t_* are the translocation rate constants for cation and anion at zero transmembrane potential, *α* is the charge transfer coefficient, *c^+^*_sp_ and *c^−^*_sp_ are the volume concentrations of cation and anion in the spacer, and *c*_0_ is the common value of the cation and anion concentrations just outside the tBLM surface. The (1 − *F*|*Г*|/45) factor is introduced to account approximately for the probability of finding free sites available for ion accommodation at the inner mouth of the ion channel; it vanishes at sufficiently negative potentials, when the cation surface charge density in the spacer, *FГ*, attains its saturation value of +45 μC·cm^−2^. The cyclic voltammetry current density *j* is exclusively controlled by ion translocation, and no ion depletion is assumed in the aqueous solution adjacent to the outer mouth of ion channels. Hence, *c*_0_ is set equal to the bulk concentration of the 1,1-valent electrolyte. The *c^+^*_sp_ value, in moles per unit volume, is obtained by dividing the moles of cations in the spacer by its volume; thus, c^+^_sp_ equals *Г^+^*/*d*, where *d* is the length of the hydrophilic spacer, estimated at 2.25 nm [53]. On the basis of analogous arguments, c^−^_sp_ is set equal to *Г^−^*/*d*. For simplicity, the potential energy barrier in the lipid bilayer is assumed to be symmetrical by setting *α* = 0.5.

Cyclic voltammetry curves are determined numerically by subdividing the potential range of interest into 1 × 10^6^ *δ*(∆*φ*) steps [19,66]. The cation current density *j*^+^ is calculated at each step via Equation (19) and integrated by adding the corresponding charge contribution, *δ*(*FГ*^+^) = *j*^+^ *δ*(∆*φ*)/*v*, to the sum of all preceding contributions, where *v* is the potential scan rate. Analogously, the anion current density *j*^−^ is calculated at each step via Equation (20) and integrated by adding the corresponding charge contribution, *δ*(−*FГ*^−^) = *j*^−^ *δ*(∆*φ*)/*v*, to the sum of all preceding contributions. The resulting *Г*^+^ and *Г*^−^ values are used to continuously update *c*^+^_sp_ = *Г*^+^/*d*, *c*^−^_sp_ = *Г*^−^/*d*, *C*_il_ via Equation (15) and *φ_m_* via Equation (18), and all these values are feedbacked into Equations (19) and (20) for the overall current density.

**Figure 16:**
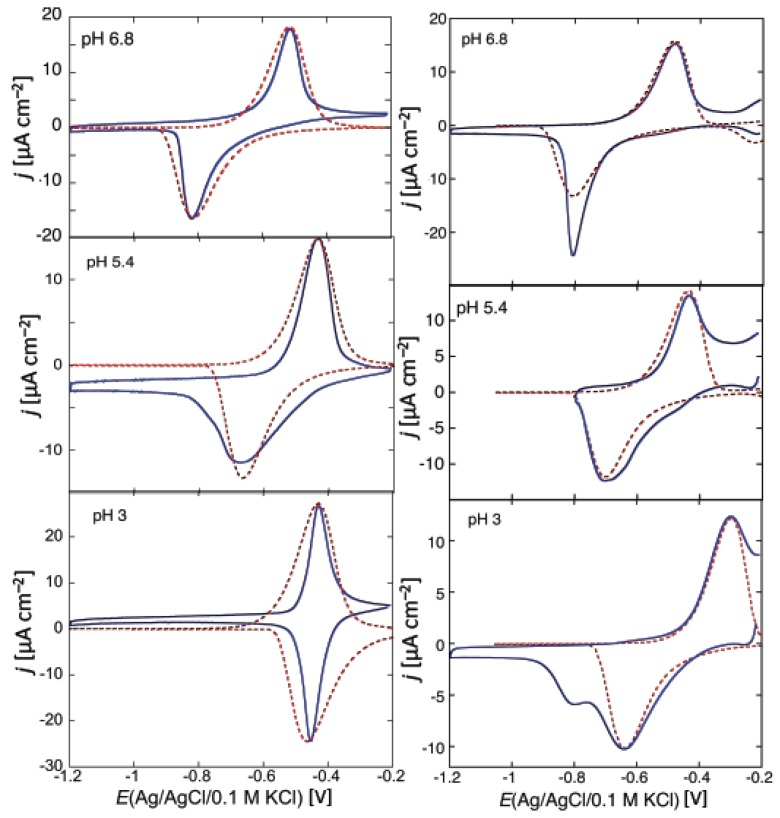
The solid blue curves are experimental cyclic voltammograms at a DPTL/DOPC tBLM in aqueous solutions of 0.1 M KCl at pH 6.8, 5.4 and 3, in the presence of either 0.1 μM gramicidin (left column) or 1 μg/mL SP25A (right column). Scan rate = 50 mV/s. The corresponding dashed curves were calculated as outlined in the text. (Reprinted from [66], Copyright 2015 and from [19], Copyright 2016, with permission from Elsevier).

The *σ_i_*/*C*_ph_ surface dipole term is ascribed to the negative charge density *σ_i_* created by the phosphate group of the DOPC polar heads surrounding the mouth of the gramicidin channel and dragged close to the hydrocarbon tail region. This charge density is expected to decrease with a decrease in pH from 6.8 to 3, due to progressive protonation of the phosphate group, but its actual value cannot be directly determined. The contrivance consisting in provisionally setting the surface dipole term *σ_i_*/*C*_ph_ in Equation (18) equal to zero at pH 6.8 is, therefore, adopted, and the focus of attention is shifted on the changes of this term with decreasing pH. With *σ_i_*/*C*_ph_ = 0, the best fit of the experimental cyclic voltammogram (CV) at pH 6.8 by the model is obtained for *k_t_*^+^ = 5 × 10^3^ cm/s and *k_t_*^−^ = 0 with the cation-selective gramicidin [66], and for *k_t_*^+^ = *k_t_*^−^ = 4 × 10^3^ cm/s with the nonselective SP25A [18,19]; the blue and red curves in the top panels of Figure 16 represent the experimental and calculated curves. The experimental changes, ∆*E_p_^n^*^(6.8→pH)^, in the negative peak potentials and those, ∆*E_p_^p^*^(6.8→pH)^, in the positive peak potentials, when passing from pH 6.8 to pH 5.4 and to pH 3, are then exclusively ascribed to corresponding changes, ∆*σ_i_*/*C*_ph_, in the surface dipole potential, and are identified with the latter, accordingly. The red curves at pH 5.4 and 3 in Figure 16 are CVs calculated from the model using the surface dipole potential changes estimated in this way, while maintaining the same *k_t_* values used for the fit of the corresponding CVs at pH 6.8. The shape of the calculated curves in Figure 16 is in fairly good agreement with that of the corresponding experimental CVs.

Most importantly, the curves calculated at the three different pH values, which are obtained from the model on the scale of the absolute potential difference ∆*φ*, retain the same relative positions along this scale as the corresponding experimental CVs do along their *E* scale, relative to the Ag/AgCl/(0.1 M KCl) reference electrode. It is, therefore, possible to superimpose the CVs calculated at the three pH values over the corresponding experimental ones by simply shifting them along their original ∆*φ* axis by the same quantity, which amounts to −130 ± 10 mV [19]. In this connection, we must consider that the absolute potential difference is more positive than the corresponding electric potential *E*, as measured vs. the Ag/AgCl/(0.1 M KCl) reference electrode, by about 0.19 V, as estimated on the basis of mild extrathermodynamic assumptions (cf. Section 3.3) [63]. Hence, the actual shift along the ∆*φ* scale required to superimpose the CVs calculated at the three different pH values over the corresponding experimental CVs on the *E* scale amounts to (−130 + 190) mV = +60 V [19].

The fact that three quite different ohmic channels, such as gramicidin, SP25A and melittin (the latter after an appropriate pretreatment [19]) require almost the same potential shift of +60 ± 10 mV to pass from the calculated CVs to the experimental ones, irrespective of pH, points to the neglect of some feature in the modelistic treatment of the tBLM. A reasonable explanation is based on the neglect of the surface dipole potentials due to both the ether linkage between the TEO spacer and the phytanyl chains and the ester linkage between the glycerol backbone and the fatty acids of the distal DOPC monolayer in the expression of Equation (17) for the absolute potential difference, ∆*φ*, across the electrified interphase. The surface dipole potential of the ester linkage is estimated at about +150 mV, positive toward the hydrocarbon tail region (cf. Section 3.3) [64]. A partial compensation of the surface dipole potential of the ester linkage by that of the ether linkage, with the sum of the two amounting to about +60 mV, might induce the small positive shift by about 60 mV, just like the surface dipole potential, *χ*_sp_ = −250 mV [63], of the TEO spacer induces a corresponding negative shift.

In spite of the similarity between the cyclic voltammetry behavior of the cation-selective gramicidin in the left column of Figure 16 and that of the nonselective SP25A in the right column of the same figure, an attentive examination of the two sets of CVs reveals a trumpet-shaped enlargement in the CVs of SP25A at the most positive potentials, which is absent in the CVs of gramicidin. A much broader enlargement is shown by the CVs of melittin, under conditions in which it behaves as an ohmic channel [19]. This is due to the same flow of chloride ions that causes an increase in the plateau of the charge transients in chronocoulometric measurements beyond the level corresponding to spacer saturation by potassium ions (cf. Section 3.1). The anion flow along nonselective ion channels at tBLMs is, however, altogether different from that predicted for a conventional BLM interposed between two bulk aqueous phases, where the anion flow is equal in magnitude and opposite in sign to the cation flow at all transmembrane potentials [38]. The anion flow at tBLMs tends to take place separately from the cation flow, at less negative potentials. More precisely, the positive peak due to anion inflow into the spacer and the negative peak due to its outflow fall at more positive potentials than the corresponding peaks for the in- and outflow of the monovalent cation (see the calculated curves in Figure 7 of [18]). This predicted behavior is due to the limited spaciousness of the spacer, which causes cations to accumulate at more negative potentials than anions. A modest overlapping of anion and cation flow is only predicted at the junction between the negative peak due to anion outflow and the negative peak due to cation inflow, and between the reverse positive peak due to cation outflow and the positive peak due to anion inflow. Unfortunately, the positive potential range required to monitor the predicted massive anion in- and outflow is experimentally inaccessible on a Hg-supported tBLM, due to mercury surface oxidation. Nonetheless, the incipient small anion inflow (during the positive-going potential scan) and its subsequent outflow (during the negative-going scan) are revealed by the trumpet-shaped enlargement exhibited at the most positive potentials by the CVs of nonselective ohmic channels.

## 4. Conclusions

Hg-supported tBLMs provide a valid alternative to conventional BLMs for the investigation of the functional activity of channel-forming peptides and small proteins. By being resistant to strong electric fields, they are capable of incorporating peptides at non-physiological negative transmembrane potentials. Even if this operation generates holes or other defects in the lipid bilayer, these are readily healed when returning to the range of physiological transmembrane potentials, where the incorporated ion channels usually maintain a satisfactory stability during subsequent electrochemical measurements. Potential-step chronocoulometry measurements allow the ion flow along ion channels to be monitored and regulated as a function of time and final potential. Cyclic voltammetry measurements allow the ion inflow into the hydrophilic spacer and its outflow to be monitored as a function of the applied potential; moreover, the rate of ion flow in and out of the spacer can be modulated by varying the potential scan rate. Forming a tBLM on a mercury cap electrodeposited on a platinum microdisk yields a micro tBLM that maintains the same fluidity and lipid lateral mobility as on a hanging mercury drop. Thus, this microdisk allows the recording of single channel currents [68,69] and of two-photon fluorescence lifetime images of lipid rafts and gel-phase microdomains in a liquid-disordered matrix [12].

In this respect, micro-tBLMs (μtBLMs) supported by a mercury film electrodeposited on platinum or iridium have the potential to be used to realize a μtBLM microarray platform for highly parallel screening of a large set of drugs and diagnostic targets on channel proteins. A sensor consisting of a Hg-coated Pt micro-cip with a phospholipid SAM on top of it was commercialized by the UK company Cymtox to detect dissolved pollutants down to the nano-scale [70]. Mercury-coated iridium ultramicroelectrode arrays have been fabricated and characterized for heavy metal trace analysis on bare Hg caps electrodeposited on the iridium micro-disks of the array [71]. These arrays benefit from the low solubility of iridium in mercury and its good wettability. Nowadays, iridium microelectrode arrays that allow the recording of the current flowing on individual micro-disks (5–10 μm in radius) are fabricated by a number of academic and industrial institutions; they are referred to as “individually addressable microarrays”. In the light of the above preconditions, the anchoring of a DPTL/lipid bilayer to all the mercury caps of a microarray in a single step can be envisioned, to ascribe exactly the same properties to all the μtBLMs of the microarray. Incorporation, say, of a very small quantity of a given membrane protein in the whole microarray would then allow a high-throughput screening of a large set of active compounds (inhibitors, promoters and modulators of the given membrane protein) in a short period of time, upon depositing the different active compounds on the different micro-disks by a robot. In view of the widespread use of DNA microarray technology, several robots are now commercially available, and could be adapted to such a membrane protein microarray. Each individual micro-disk of the array would then be addressed and interrogated by an electrochemical technique, using a multichannel electrochemical detection system. By far the most suitable technique is electrochemical impedance spectroscopy, although potential step chronoamperometry and chronocoulometry might come in handy under particular circumstances for the activation of the integral protein under study. Such a technological platform has the potential to be developed into commercial products solving high throughput needs for user groups in the academic and commercial sectors in the fields of life science, biotechnology and analytical chemistry.
